# Branching Ionizable Lipids Can Enhance the Stability, Fusogenicity, and Functional Delivery of mRNA

**DOI:** 10.1002/smsc.202200071

**Published:** 2022-11-09

**Authors:** Kazuki Hashiba, Yusuke Sato, Masamitsu Taguchi, Sachiko Sakamoto, Ayaka Otsu, Yoshiki Maeda, Takuya Shishido, Masao Murakawa, Arimichi Okazaki, Hideyoshi Harashima

**Affiliations:** ^1^ Nucleic Acid Medicine Business Division Nitto Denko Corporation 1-1-2, Shimohozumi Ibaraki Osaka 567-8680 Japan; ^2^ Laboratory for Molecular Design of Pharmaceutics, Faculty of Pharmaceutical Sciences Hokkaido University Kita-12, Nishi-6 Kita-Ku Sapporo 060-0812 Japan

**Keywords:** branched tail, genome editing, ionizable lipids, lipid nanoparticles, mRNA

## Abstract

Ionizable lipids with branched tails have been used in lipid nanoparticles (LNPs)‐based messenger RNA (mRNA) therapeutics like COVID‐19 vaccines. However, due to the limited commercial availability of branched ingredients, a systematic analysis of how the branched tails affect LNP quality has been lacking to date. Herein, α‐branched tail lipids are focused, as they can be synthesized from simple commercially available chemicals, and the length of each chain can be independently controlled. Furthermore, symmetry and total carbon number can be used to describe α‐branched tails, facilitating the design of a systematic lipid library to elucidate “structure–property–function” relationships. Consequently, a lipid library is developed containing 32 different types of α‐branched tails. This library is used to demonstrate that branched chains increase LNP microviscosity and headgroup ionization ability in an acidic environment, which in turn enhances the stability and in vivo efficacy of mRNA‐LNPs. Of the branched lipids, CL4F 8‐6 LNPs carrying Cas9 mRNA and sgRNA could achieve 54% genome editing and 77% protein reduction with a single dose of 2.5 mg kg^−1^. This mechanism‐based data on branched lipids is expected to provide insights into rational lipid design and effective gene therapy in the future.

## Graphical abstract

Branched‐tail lipids are commonly used for the delivery of messenger RNA (mRNA). However, the exact role of the branched chains remains unclear. To address this, a lipid library is developed containing 32 different types of α‐branched tails. This library enables a systematic comparison and demonstrates that branched chains can enhance the stability, fusogenicity, and functional delivery of mRNA.

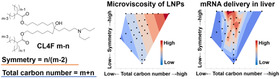

## 1 Introduction

Advances in messenger RNA (mRNA) technology have accelerated a wide range of mRNA therapeutic applications, including infectious disease vaccines, cancer immunotherapies,^[^ ^1^, ^2^ ^]^ allergy tolerization,^[^ ^3^ ^]^ protein replacement therapies, genome engineering, and genetic reprogramming.^[^ ^4^, ^5^ ^]^ Notably, mRNA vaccines were officially approved in response to the COVID‐19 pandemic and they contributed to a significant decrease in the resulting mortality rate.^[^ ^6^, ^7^ ^]^ Despite the great potential of mRNA for further pharmaceutical applications, their intracellular delivery remains a challenge due to their large molecular weight, polyanionic nature, and inherent chemical instability.

Lipid nanoparticles (LNPs) are one of the most advanced technologies available for the efficient in vivo delivery of exogenous mRNAs. They are typically composed of ionizable lipids, cholesterol (chol), helper lipids, and polyethylene glycol (PEG) lipids, and they are responsible for the inhibition of mRNA degradation and transport across the plasma membrane into the cytosol. Ionizable lipids are key components of most LNPs as they can encapsulate mRNAs through electrostatic interactions. At a physiological pH, a neutral charge improves the pharmacokinetics in the body, while at an acidic pH, protonated lipids promote fusion with endosomal membranes and the release of mRNA into the cytosol.

The head and tail groups of typical ionizable lipids have different roles. The headgroup is the positively charged moiety and often has a tertiary amine, of which there are a wide range of types, such as alkyl and cyclic amines.^[^ ^8^ ^]^ The headgroup determines the apparent pKa of the LNPs, which regulates their fate in vivo. In contrast, the lipid tail is the hydrophobic moiety, responsible for particle formation. Unsaturated tails,^[^ ^9^ ^]^ biodegradable tails,^[^ ^10^, ^11^ ^]^ polymeric tails,^[^ ^12^, ^13^ ^]^ and branched tails^[^ ^14^, ^15^ ^]^ have all previously been investigated.

Focusing on the branch tail lipids, several groups have reported that branched tails are beneficial for the delivery of mRNA. For example, Dong et al. found out that branched lipids could achieve effective transfection in vitro when compared to linear lipids.^[^ ^16^ ^]^ Hajj et al. also reported that an isodecyl (branch at the end) tail is 10‐fold more active in vivo than a linear tail.^[^ ^17^ ^]^ In clinical studies, branched tail lipids have been used in COVID‐19 vaccines and genome editing with Cas9 mRNA delivery.^[^ ^18^ ^]^ These studies have provided important insights, but the comparison of only a few lipids does not equate to a systematic understanding of branched tail lipids.

In this study, to better understand the impact of the branched tail structure on the function of mRNA‐LNPs, a systematic branched lipid library was generated. We showed detailed comparisons of 32 different branched tail lipids and we have demonstrated that the branched chains of the tail enhance the stability and in vivo efficacy of the mRNA‐LNPs. Of the lipids with branched chains, the CL4F 8–6 LNPs complexed with Cas9 mRNA and sgRNA could achieve efficient genome editing after a single administration. The systematic data demonstrate the advantage of branched lipids and are expected to help direct rational lipid design in the future.

## 2 Results and Discussion

### 2.1 Development of a Systematic Lipid Library to Investigate Branched Tails

The branching structure creates a large chemical space. Consequently, branched compounds have many structural parameters such as the number and position of branched chains, the length of each hydrocarbon chain, the symmetry of the main and branched chains, the total carbon number, and the number of heteroatoms. This complexity has previously prevented accurate comparisons among these branched compounds. The preparation of a rationally designed lipid library has also been difficult due to the limited number of commercially available branched chemicals. Thus, there remains a need to improve our understanding of branched chains and how they affect the quality of mRNA‐LNPs.

In this investigation, we have focused on the α‐branched tail lipid and reduced its structural descriptors to two simple parameters: symmetry and total carbon number. α‐branched fatty acids can also be synthesized from simple commercially available chemicals, and the length of each chain can be independently controlled, which is ideal for the design of a systematic lipid library to assess a structure–activity relationship (SAR). Here, we have generated an α‐branched tail lipid library in which the head structure is the same, but the α‐branched tails have 32 different structural diversities.

Alkylating the α‐carbon of the carbonyl compounds using enolates yields α‐branched fatty acids. Utilizing two different synthetic routes depending on the difficulty of purification, targeted α‐branched tails were obtained by separating them from the raw materials and unintended byproducts. One route for this is the alkylation of dimethyl malonate with one or two iodoalkanes (C1–C15), the other is the alkylation of linear fatty acids with iodoalkanes.

**Figure 1:**
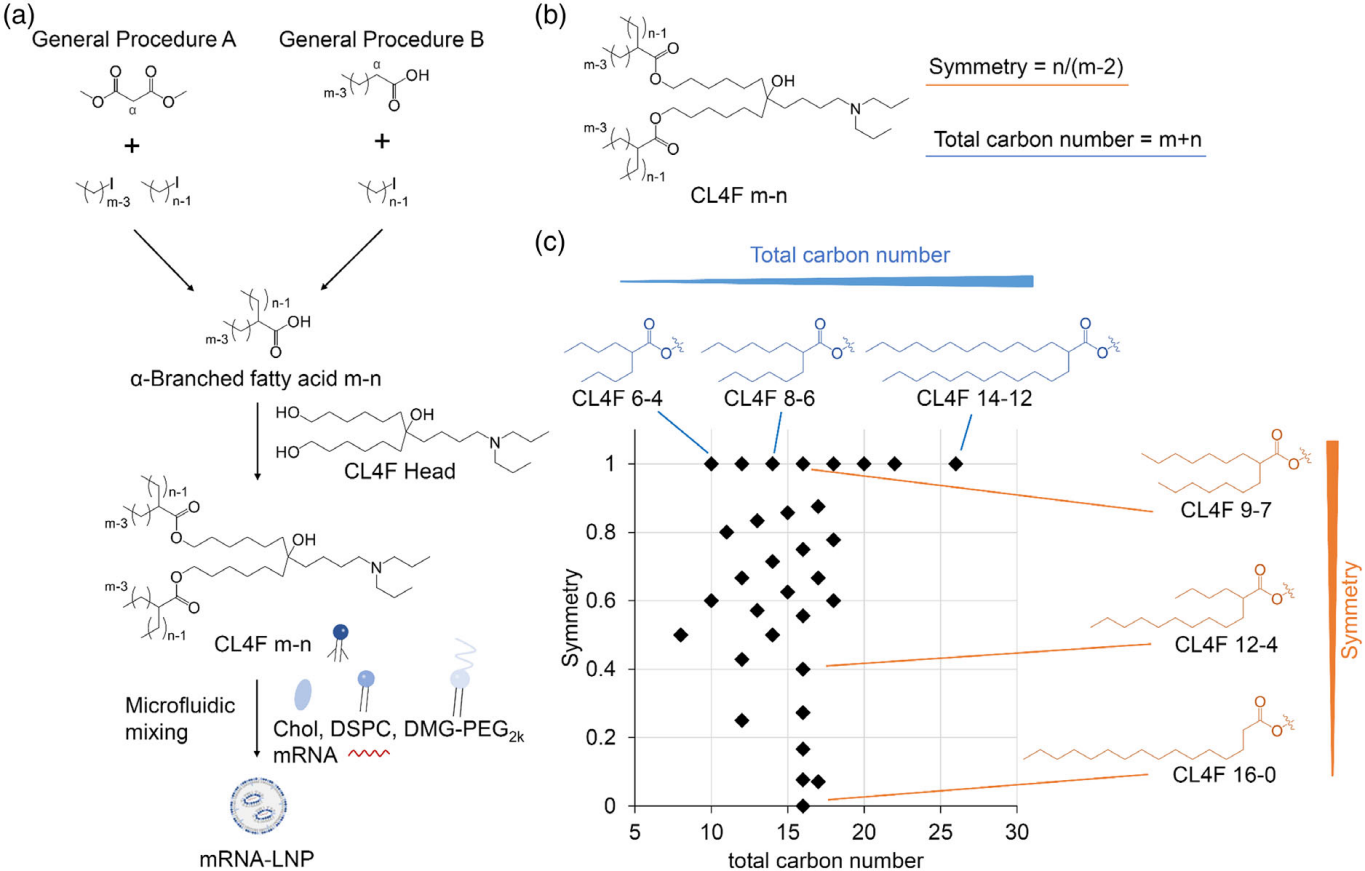
A branched lipid library based on sequential symmetry and total carbon number was constructed. a) α‐Branched fatty acids were synthesized according to General Procedure A or B. CL4F *m* − *n* was synthesized by the esterification of fatty acids *m* − *n* with an aminoalcohol CL4F head; “*m*” in “CL4F *m* − *n*” indicates the length of the alkyl main chain; “*n*” indicates the length of the alkyl side chain. LNPs were formulated by mixing ionizable lipid, cholesterol, DSPC, and DMG‐PEG2k (50:38.5:10:1.5 mol%) with mRNA using the ethanol dilution method. b) Each lipid was described using two characteristic parameters: total carbon number and symmetry. The total carbon number of each tail is *m* + *n*, and these ranged from C8–C26. The symmetry of the main chain and side chain is represented as the ratio of *n* to *m* − 2. c) There were 32 α‐branched‐tail lipids generated with independently different alkyl lengths in the main and side chains, and these were used to construct the lipid library. Each lipid was plotted according to its total carbon number and symmetry.

The headgroups of the novel lipid library members were fixed with a CL4 structure, which is an ionizable lipid that was previously developed.^[^ ^19^ ^]^ The lipids were synthesized by the esterification of α‐branched fatty acids (termed *m* − *n*, where “*m*” is the main chain length and “*n*” is the side chain length), and they yielded α‐branched‐tail lipids (termed CL4F *m* − *n*) (**Figure** **1**a).

Ionizable lipids were purified using both normal‐phase and reversed‐phase chromatography. The lipids were all identified using proton nuclear magnetic resonance (^1^H‐NMR) and positive mode liquid chromatography with mass spectrometry (LC/MS). The purity was measured by liquid chromatography with charged aerosol detection (LC/CAD) and confirmed to be over 83.9%.

The α‐branching tail is described based on its symmetry and total carbon number (Figure 1b). The symmetry of the main chain and side chain is calculated as the ratio of *n* to *m* − 2. The total carbon number for the α‐branched tail was calculated as *m* + *n* and it can range from C8–C26. Each of the 32 lipid types assessed was plotted according to their tail structures (Figure 1c). For example, CL4F 9–7, CL4F 10–6, CL4F 12–4, and CL4F 16–0 have the same total carbon number (16) in each tail, but their symmetry (*y*‐axis) varied, and were fully symmetrical (1.0), highly symmetrical (0.75), moderately symmetrical (0.4), and linear (0.0), respectively. In contrast, CL4F 6–4, CL4F 9–7, and CL4F 14–12 have fully symmetrical (1.0) branched tails, but the total carbon number for their tails (x‐axis) varied, and was 10, 16, and 26, respectively. This simple description of the structure facilitates an improved understanding of the “structure–property–function” relationships among the branched‐tail lipids.

### 2.2 Impact of Tail Chemistry on the mRNA‐LNP Formulation

LNPs were formulated by mixing ionizable lipids, cholesterol, 1,2‐distearoyl‐sn‐glycero‐3‐phosphocholine (DSPC), and 1,2‐dimyristoyl‐rac‐glycero‐3‐methoxypolyethylene glycol‐2000 (DMG‐PEG2k) (50:38.5:10:1.5 mol%) with mRNA using an ethanol dilution method. Despite the same composition and formulation process, the physicochemical properties of the LNPs immediately after preparation varied depending on the tail structure of the ionizable lipids (Table S1, Supporting Information). Particle size ranged from 65–220 nm, the polydispersity index (PDI) from 0.002–0.225, and the encapsulation efficiency from 40% to 100%.

The apparent pKa of the LNPs helps to explain their in vivo fate and it was reported to shift dynamically if the head structure changed.^[^ ^9^ ^]^ However, despite the 32 ionizable lipids in the constructed library having the same headgroup, the apparent pKa values were found to range from 5.85 to 6.81. This indicates that not only the head but also the tail structure contributes to the apparent pKa.

**Figure 2:**
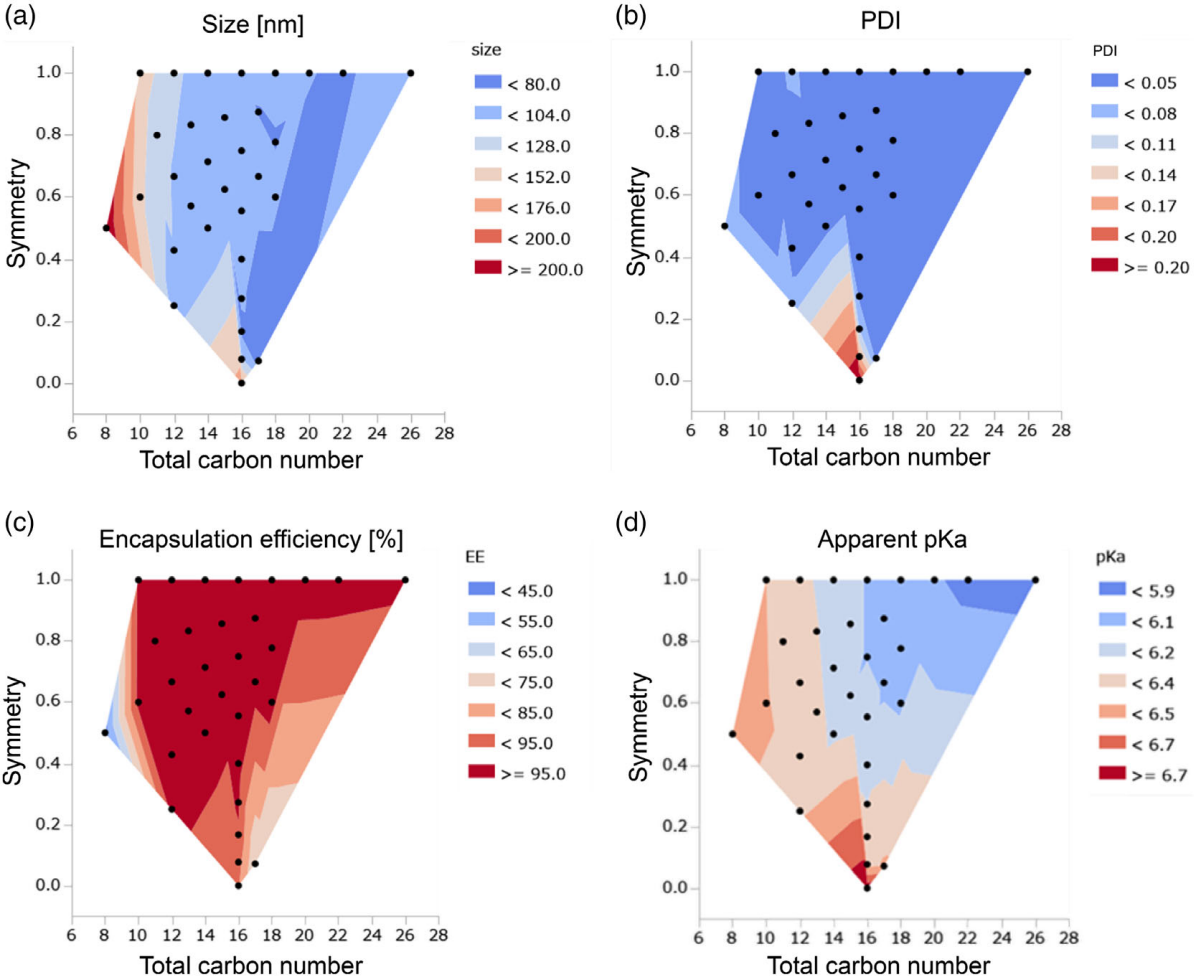
α‐Branched tail structures affect the physicochemical characteristics of the LNPs. Physicochemical characteristics measured immediately after LNP formulation were plotted on contour plots, where the *x*‐ and *y*‐axis represent the total carbon number and symmetry, respectively. a) ζ‐Average, b) polydispersity index (PDI), c) mRNA encapsulation efficiency, and d) apparent pKa of LNPs.

To visualize the influence of tail chemistry on mRNA‐LNP formulations, contour plots were prepared with the two parameters describing the branched structure, symmetry, and total carbon number (**Figure** **2**a–d). The increase in total carbon number contributes to the smaller particle size with narrow PDI and higher encapsulation efficiency, probably due to the hydrophobicity of the lipids as indicated by the log*P* value. It was shown that higher symmetry contributes to well‐controlled particle size with higher encapsulation efficiency. However, this tendency cannot be explained by the hydrophobicity of lipids and it is hard to understand the mechanism. Figure 2d suggests that there is a regular pattern of change in pKa values related to the tail structure, but the underlying mechanism is unclear. Currently, there are very limited studies on how their physicochemical properties are controlled by the chemical structure of the lipids. For example, M. Cornebise et al. suggested that chemical structure influences the size and heterogeneity of LNPs via head‐mRNA interplay.^[^ ^20^ ^]^ This study will guide future lipid design but was mainly a comparison of head structures. Therefore, we tried to perform a mechanism‐based study of how tail chemistry impacts physicochemical properties.

### 2.3 Microviscosity in LNPs Increased Due to Branched Chains

**Figure 3:**
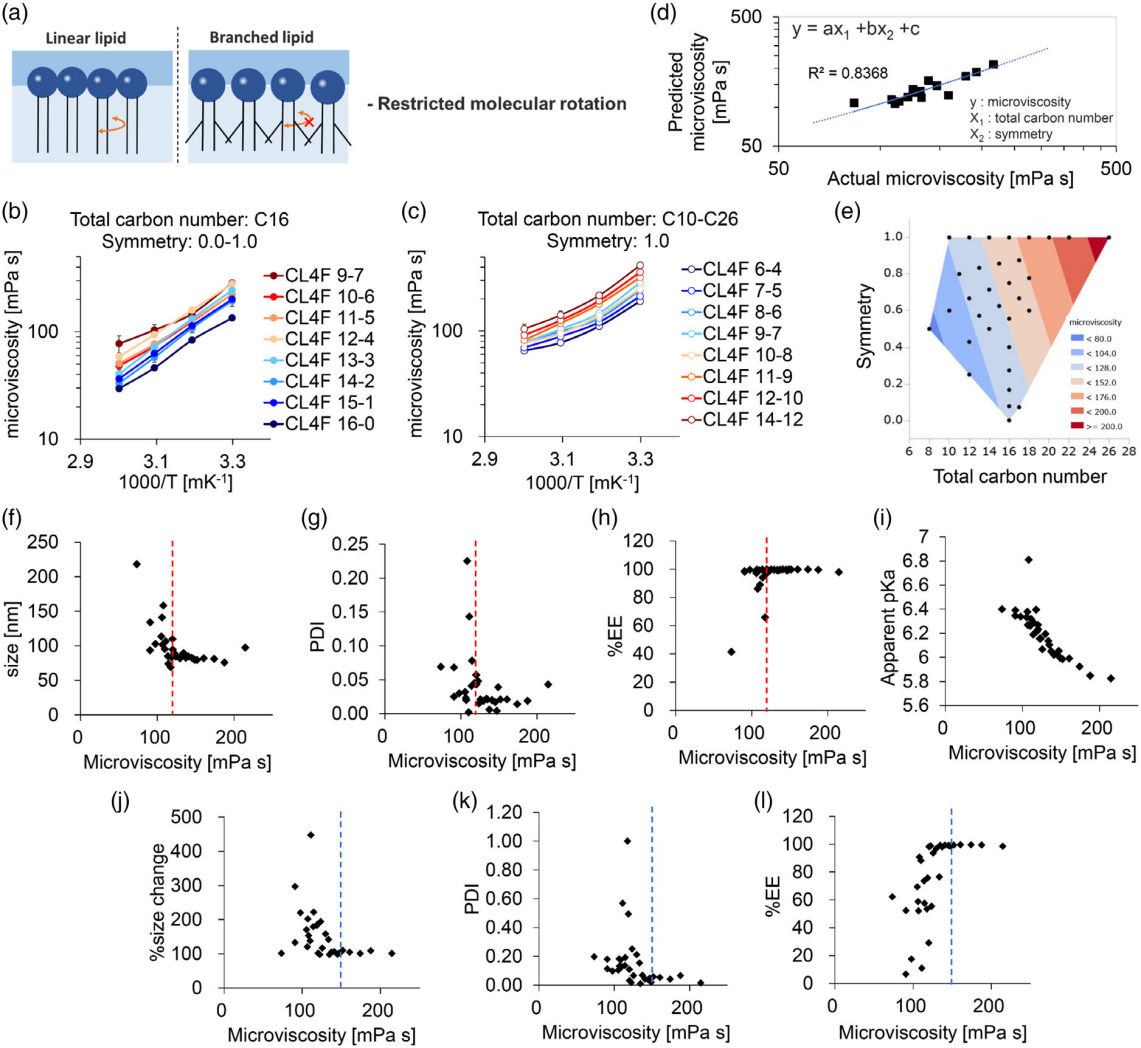
Branched chains increase the microviscosity of LNPs, which contributes to the physical stability of mRNA‐LNPs. a) The branched side chains of the lipid tails are known to limit molecular rotation in the lipid membrane. b) 9‐(Dicyanovinyl)‐julolidine (DCVJ)‐labeling experiments quantified the microviscosity of eight lipid nanoparticles (LNPs) containing ionizable lipids with the same total carbon number (C16) but different symmetries (0.0–1.0), *n* = 3. c) The microviscosity of eight LNPs containing fully symmetric (1.0) ionizable lipids with different total carbon number within the range C10–C26, *n* = 3. d) A multiple regression was performed using the total carbon number and symmetry patterns. The higher correlation coefficient (*R* ^2^ = 0.8368) suggests that the microviscosity at 40 °C (*y*) depends on the total carbon number (*x*_1_) and symmetry (*x*_2_). e) For the 32 types of lipids, microviscosity was estimated from the chemical structures and plotted against the symmetry and total carbon number of the branched tails. f–h) The regressed microviscosity values were plotted against their physicochemical properties immediately after preparation. The LNPs with a microviscosity >120 mPa s (red dashed lines) showed smaller particle sizes with a narrow PDI and improved encapsulation efficiency. i) Regressed microviscosity values were related to the apparent pKa. j–l) The physicochemical properties of each LNPs were assessed after 30 d of being stored at 40 °C. The LNPs with viscosity <150 mPa s (blue dashed lines) had an increased particle size with a higher PDI and leaked mRNA.

Hydrophobic tails play an important role in particle formation, as their hydrophobicity is the driving forces behind self‐assembly.^[^ ^21^ ^]^ To understand the impacts of branched tail chemistry on the physicochemical properties of LNPs, we focused on the relationship between the molecular conformation and rotational motion of the lipids in LNPs. Several in silico studies have suggested that branched chains limit the rotational motion of the molecule in the lipid bilayer, as indicated by an increase in the areas elastic moduli and the axial relaxation time of the lipids.^[^ ^22^, ^23^ ^]^ On a molecular level, a high gauche probability due to the branched chains brought about chain bending and the slow conformational motion of the hydrophobic chain (**Figure** **3**a).^[^ ^24^ ^]^ Taking this into account, we hypothesized that the restriction of the molecular rotation by the branched chain would stabilize the nanoparticles, and this would result in a smaller particle size with lower PDI and higher encapsulation efficiency.

To experimentally evaluate the role of the branched chains on the LNPs, we have focused on microviscosity, which is important for membrane transport, membrane‐bound enzyme function, and receptor binding. Viscosity on the macroscale is typically measured by mechanical methods, while at the microscale it can be monitored using fluorescent probes or markers. Fluorescent techniques include single molecule tracking,^[^ ^25^ ^]^ fluorescence recovery after photodegradation,^[^ ^26^ ^]^ fluorescence correlation spectroscopy (FCS),^[^ ^27^ ^]^ and fluorescence anisotropy.^[^ ^28^ ^]^ Among the techniques used to measure microviscosity, the molecular rotor is one of the most powerful.^[^ ^29^, ^30^ ^]^ The molecular rotor is a fluorescent probe based on a twisted intramolecular charge transfer (TICT), in which the quantum yield and fluorescence lifetime both respond to microviscosity. This mechanism enables molecular rotors to respond to nano‐scale viscosities with higher sensitivity. Of the molecular rotors, Push–pull type molecular rotors such as 9‐(dicyanovinyl)‐julolidine (DCVJ) and 9‐(2‐carboxy‐2‐cyano)vinyljulolidine (CCVJ) and BODIPY‐based molecular rotors such as BODIPY‐C_12_ are widely used for monitoring microviscosity. DCVJ, which is not chargeable, was selected to eliminate the influence of electrostatic interactions of the molecular rotor with ionizable lipids on the measurement values. The intramolecular rotation of DCVJ occurred around the single bond between the julolidine and dicyanovinyl groups. This caused nonradiative thermal relaxation of the excitation energy, resulting in the quenching of the fluorescence intensity (Figure S1a, Supporting Information).^[^ ^31^ ^]^ The DCVJ has been shown to be predominantly partitioned in the lipid membrane and detects differences in microviscosity between DPPC/cholesterol liposomes and DOPC/cholesterol liposomes.^[^ ^32^ ^]^ Typically, LNPs are formed from a core which contains mRNA, water, ionizable lipid, and cholesterol, as well as a DSPC‐enriched layer and a PEG layer.^[^ ^33^ ^]^ The hydrophobic nature of DCVJ allows it to locate in the hydrophobic region of LNPs, making it suitable for microviscosity measurements of internal LNPs as well as the liposome lipid membrane.

First, we confirmed that the fluorescence intensity from DCVJ strongly responds to viscosity in the glycerol/methanol mixtures (Figure S1b, Supporting Information). In contrast, the DCVJ absorption spectrum was consistently independent of the viscosity and could thus be used to correct the dye concentration (Figure S1c, Supporting Information). To convert measured values into viscosity, we utilized an integrated fluorescence emission and absorbance ratio (denoted as “R”) instead of the quantum yield.^[^ ^32^ ^]^ When the measured R values were plotted against the viscosity values of the glycerol/methanol mixture at each temperature (30–60 °C), it enabled the fitting of a calibration curve based on the power law (*R* ^2^  = 0.833) (Figure S1d, Supporting Information). The value of the exponent (0.581) determined by fitting the power law is within the range (0.51–0.59) of previous reports.^[^ ^34^, ^35^ ^]^

Next, we measured the R values for each of the 15 LNP types labeled with DCVJ at each temperature (30–60 °C) and converted them into microviscosity. When the microviscosity of each LNP was plotted against temperature, a data profile could then be fitted using the Arrhenius law within the range of 30.3–50.3 kJ mol^−1^ as activation energy, which is consistent with a previous study.^[^ ^32^ ^]^ No transition point was observed since 38.5% cholesterol prevents a rapid phase transition. Considering the absence of a transition point and its association with the thermostability data, the microviscosity of each LNP was compared at 40 °C.

We compared the microviscosity of 8 different LNPs prepared from each ionizable lipid with the same carbon number (C16) but different symmetries (0.0–1.0) (Figure 3b). The results showed that highly symmetric lipids, i.e., those with longer side chains, tended to form more viscous LNPs. For example, CL4F 9–7 (longer side chain) and CL4F 14–2 (shorter side chain) had 1.76‐ and 1.29‐fold increases in microviscosity, respectively, when compared with the linear lipid CL4F 16–0 at 40 °C. Longer side chains are thus thought to restrict the rotational motion of lipid molecules. In addition, we compared the microviscosity of eight different LNPs prepared from fully symmetric ionizable lipids with different total carbon numbers (C10–C26) (Figure 3c). The results confirmed that lipids with a higher total carbon number tended to form more viscous LNPs when compared to CL4F 6–4 (C10) at 40 °C. For example, CL4F 14–12 (C26) and CL4F 9–7 (C16) were 1.96‐fold and 1.47‐fold higher in microviscosity, respectively, due to their increased hydrophobicity.

Thus, microviscosity follows a highly ordered pattern depending on the branched chemical structure (symmetry and total carbon number), which was regressed using multiple regression analysis (*R* ^2^  = 0.837) (Figure 3d). Furthermore, the factor analysis results made it clear that both symmetry and total carbon number significantly contribute to microviscosity (Table S2, Supporting Information). For 32 types of lipids, regressed microviscosity was visualized on a contour map (Figure 3e). This figure clearly illustrates that both symmetry and total carbon number contribute to increases in microviscosity. When the regressed microviscosity values were plotted against each of the physicochemical characteristics, a clear trend was identified as the LNPs with higher microviscosity had smaller particle sizes with lower PDI and improved encapsulation efficiency (Figure 3f–h). When the microviscosity falls below 120 mPa s, the deterioration of each physicochemical property is observed.

Surprisingly, a strong correlation was also found between microviscosity and the apparent pKa of the LNPs (Figure 3i). This is consistent with our previous study in which branched tails (assumed to be viscous) were found to cause a decrease in pKa when compared to the linear tails.^[^ ^19^ ^]^ The apparent pKa was strongly influenced by the local dielectric constant as the ionizable head group is located at the lipid membrane.^[^ ^36^, ^37^ ^]^ It was thus speculated that more viscous LNPs may require a higher proton concentration to ionize, as the amine headgroups are less likely to escape the charge repulsion in the viscous membrane.

### 2.4 Relationship Between the Microviscosity and Physical Stability of the LNPs

The storage stability of the mRNA‐LNPs is an important issue. For mRNA‐LNPs to remain functional after storage, the chemical degradation of mRNA and lipids, as well as the physical degradation of LNPs, must be prevented. Chemical degradation can result in mRNAs losing their activity due to hydrolysis,^[^ ^38^ ^]^ oxidation, and transesterification.^[^ ^39^, ^40^, ^41^ ^]^ Furthermore lipids can undergo hydrolysis or oxidation.

To prevent physical degradation, it is recognized that heating and repeated freeze–thaw cycles should be avoided, but the impacts of lipid chemical structures on LNP stability are poorly understood. As microviscosity is related to energy that inhibits the lipids attempting to diffuse freely within LNPs, we have speculated that microviscosity will affect the physical stability of LNPs as well as their physicochemical properties immediately after preparation.

To investigate these relationships, LNPs were stored at various temperatures (4, 25, or 40 °C) and their physicochemical characteristics were assessed for up to 90 d (Table S3, Supporting Information). As expected, less viscous LNPs such as CL4F 15–1 and CL4F 14–2 gradually increased in particle size and polydispersity and over time, leaked mRNA. To better clarify the impacts of microviscosity on stability, microviscosity was plotted against physicochemical properties after 1 month of storage at 40 °C (Figure 3j–l). The results showed that LNPs with microviscosity below ≈150 mPa s have poor storage stability. These data suggest that the lipid tail structure determines the properties of LNPs, and thus influences their physical stability.

### 2.5 Maximizing the Ionization Ability of Amine Headgroups and Endosomal Escape Efficiency as a Function of the Space Generated by Branched Chains

**Figure 4:**
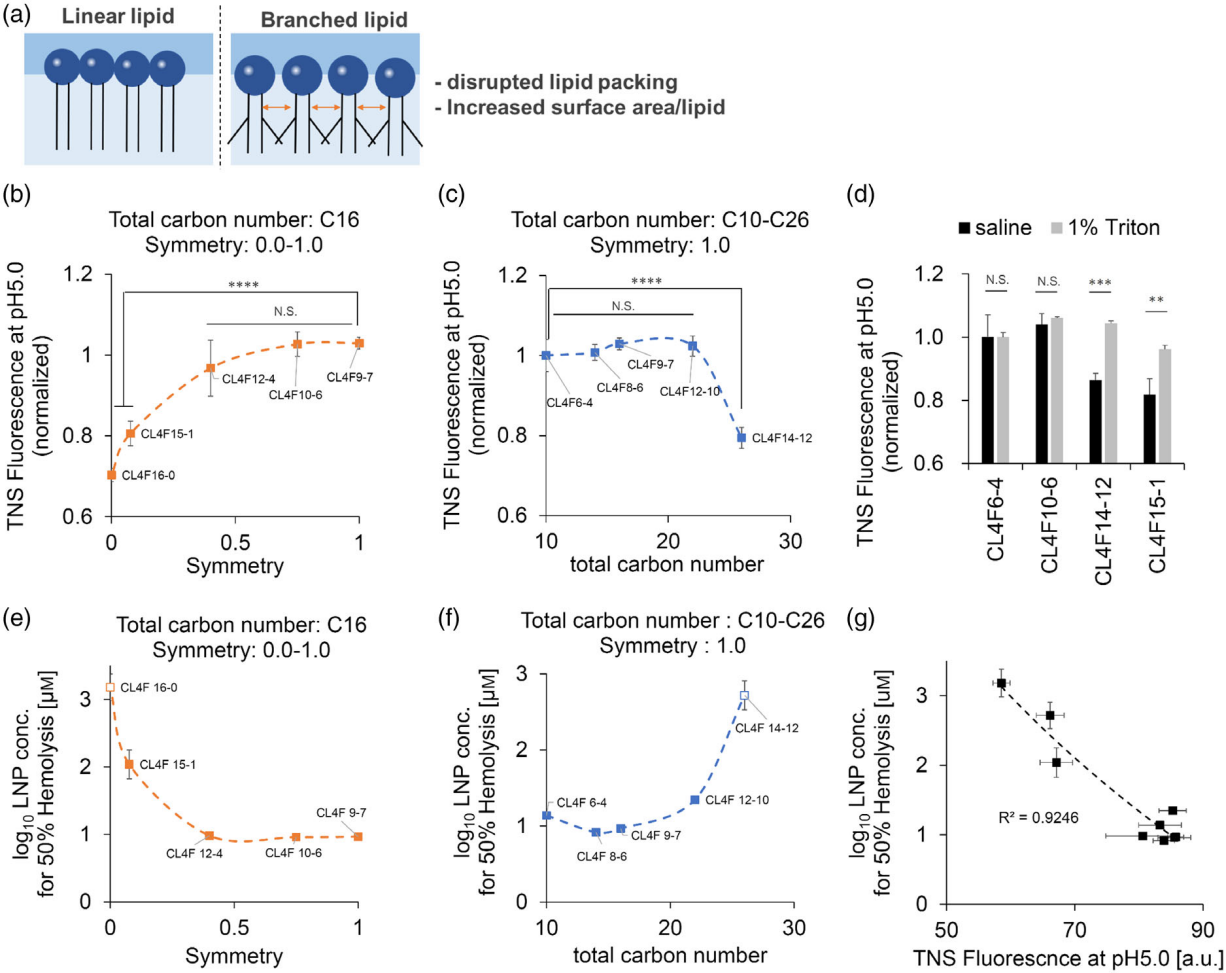
Branched side chains maximize the ionization ability of amine headgroups and endosomal escape efficiency by generating additional space. a) The branched‐chain of the lipid tail is known to disrupt lipid packing in the membrane and increase the surface area of each lipid. b) A 6‐*p*‐toluidino‐2‐naphthalenesulfonic acid (TNS) assay revealed the ionization ability of 5 LNPs that contain ionizable lipids with the same carbon number (C16) but different symmetry (0.0–1.0), *n* = 3. c) The ionization ability of five fully symmetrical lipids with the different carbon numbers (C10–C26), *n* = 3, **** *P* < 0.0001, determined using one‐way ANOVA followed by Dunnett's post‐tests for comparison of multiple groups (b) versus CL4F 9–7. b) versus CL4F 6–4. N.S.: not significant. d) LNPs were solubilized with TritonX‐100 to create space for ionization. No significant differences were detected in the CL4F 6–4 and CL4F 10–6, while lipids with a low ionization ability, such as CL4F 14–12 and CL4F 15–1, had an improved TNS fluorescence at pH 5.0. This suggests that the tightly packed conformation that originated from the tail structures hindered ionization, *n* = 3, ** *P* < 0.01 and *** *P* < 0.001, determined using the unpaired Student's *t*‐test. N.S.: not significant. e–f) The LNP concentration at which 50% hemolysis occurs according to the curve fitting. Data are represented as the mean ± standard error. LNPs that did not induce more than 50% hemolysis relative to the positive control, even at 250 μm, were extrapolated with a fitting curve to estimate 50% hemolysis and were represented by open squares, *n* = 3. g) Ability of ionization to regulate hemolysis activity.

After the cellular uptake of LNPs, ionizable lipids are responsible for the release of mRNA into the cytoplasm as they facilitate membrane fusion through protonation upon endosomal acidification. Hajj et al. experimentally suggested that the methyl branch at the end of the hydrophobic tail enhanced the ionization ability at pH 5.0 which was correlated with in vivo efficacy.^[^ ^17^ ^]^ Based on an in silico approach, branched chains are known to disrupt lipid packing, as indicated by an increase in surface area per lipid and a decrease in lipid chain order (**Figure** **4**a).^[^ ^22^, ^23^ ^]^ Shinoda et al. produced a simulation to show that the branched lipid DPhPC was packed in a more disrupted manner than the linear DPPC, due to chain bending.^[^ ^24^ ^]^ Overall, we have hypothesized that the additional space created by the branched‐chain maximizes the ionization capacity. The branched tails in the lipid library that was generated are a variety of carbon lengths and thus an ideal tool with which to investigate the aforementioned hypothesis.

We compared the ionization ability of five lipids with different lengths of the branched chain (linear or methyl branched ‐ heptyl branched) by utilizing the anionic fluorescent dye 6‐*p*‐toluidino‐2‐naphthalenesulfonic acid (TNS). To eliminate the influence of the total carbon number, each lipid tail used had the same total carbon (C16), but different symmetry. Consequently, the methyl‐branched CL4F 15–1 was found to have a higher ionization ability when compared to the linear CL4F 16–0, which is consistent with an earlier study on the methyl‐branched lipid (Figure 4b).^[^ ^17^ ^]^ Additionally, we have gained a new understanding that the longer the branched side chain, the more effective its ionization (Figure 4b). This is possible because longer side chains generate more additional space between the lipid molecules and maximize the protonatable capacities of the amine headgroups. We further analyzed five fully symmetric lipids with different total carbon numbers (C10–C26). Almost all the fully symmetric lipids, except CL4F 14–12 (C26), were found to have a high ionization capacity due to their long side chains (Figure 4c). CL4F 14–12, which has the highest total carbon number in our lipid library, only had 80% protonation when compared to CL4F 6–4, and this may be because strong van der Waals interactions have filled the space.^[^ ^42^, ^43^ ^]^

To further support the hypothesis that the space created by the branched tails maximizes the ionization ability, tightly packed LNPs such as CL4F 14–12 and CL4F 15–1 were solubilized with a surfactant to create the space. To avoid the influence of charge on the assay, TritonX‐100, as a nonionic surfactant was added to the LNPs and the ionization ability at pH 5.0, was measured using TNS. The addition of TritonX‐100 improved the ionization in CL4F 14–12 and CL4F 15–1, which was expected, while for CL4F 6–4 and CL4F 10–6 it remained unchanged (Figure 4d). This data strongly suggests that the tightly packed conformation of the LNPs restricts the protonation of ionizable lipids.

To investigate whether maximizing the ionization ability of the amine headgroups could contribute to endosomal escape, the membrane‐disrupting activity was evaluated using a hemolysis assay in an acidic environment. Different concentrations of the LNPs were incubated with red blood cells (RBCs) to estimate the concentration at which 50% hemolysis was induced. As expected, lipids with a higher symmetry and smaller total carbon number induced 50% hemolysis at concentrations ≤10 μm (Figure 4e,f). Furthermore, a strong correlation was confirmed between ionization ability and hemolysis at pH 5.0 (Figure 4g). Hemolysis activity and ionization under physiological pH were so low that there is little need to consider nonspecific interactions in the blood (Figure S2b–d, Supporting Information). These data have demonstrated that branched side chains disrupt tightly packed membranes, which creates space for protonation in acidic environments and thus facilitates endosomal escape. It is important to note that the chain length itself of the lipids may also affect endosomal escape in terms of adaptation in the cellular lipid bilayer. Thus, the low hemolysis activity of CL4F 14–12 and CL4F 16–0 may be attributed not only to their low level of ionization ability but also to their low adaptation in the cellular lipid bilayer with too long chain length.

### 2.6 “Structure–Property–Function” Relationships for In Vivo mRNA Delivery

**Figure 5:**
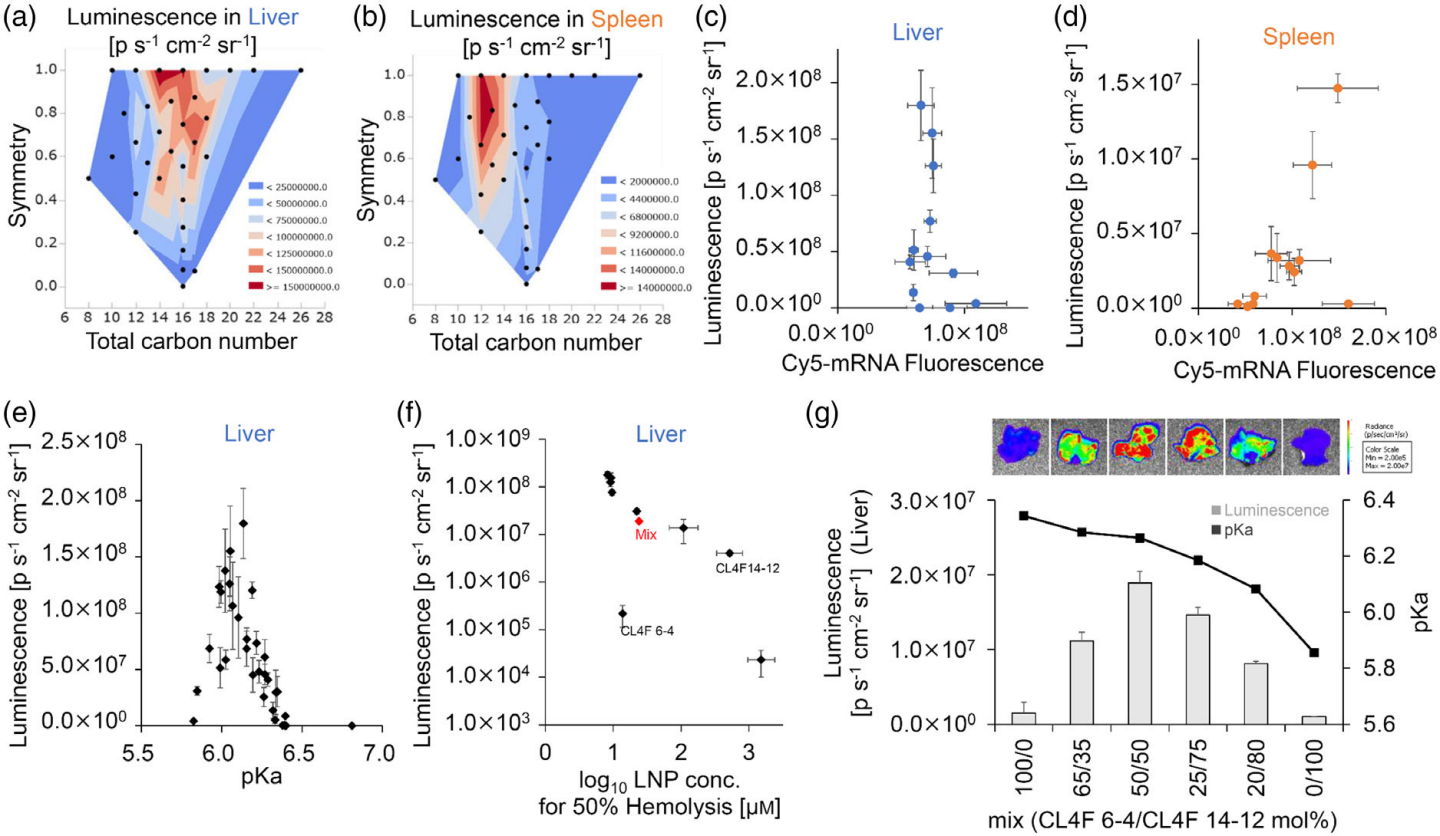
“Structure–property–function” relationships for in vivo mRNA delivery. a,b) Ex vivo bioluminescence in the liver and spleen was measured 6 h after the administration of the 32 different types of LNPs (0.1 mg kg^−1^ Fluc mRNA). Each value was plotted on the contour plots, where the *X* and *Y* axis are the total carbon number and symmetry. The bioluminescence varied dramatically depending on the tail structure. *n* = 3 c,d) LNPs containing 12 representative ionizable lipids loaded with Cy5‐mRNA (0.5 mg kg^−1^ Cy5 labeled Fluc mRNA) were administered and their biodistribution was evaluated using IVIS 3 h after administration. When the biodistribution was plotted against the mRNA expression, no correlation was observed in the liver, while accumulation was correlated with expression in the spleen except for CL4F 6–4. e) pKa is apparently a useful parameter by which to predict in vivo efficacy in the liver, but it alone cannot explain the approximately10‐fold difference in luminescence observed in the optimal pKa range. f) Hemolytic activity in an acidic environment was also correlated with expression in the liver except for CL4F 6–4. Red diamonds indicate mixed (CL4F 6–4/CL4F 14–12 = 50:50 mol%) LNPs. g) In vivo evaluation of mixed LNPs at 0.1 mg kg^−1^ Fluc mRNA. Upper panel: bioluminescence of the liver was captured 6 h after administration. Lower panel: An increase in the ratio of short‐tail lipids was attributed to higher pKa values. The mixed LNPs resulted in a tenfold increase in the efficiency of delivery when compared with each LNP alone. *n* = 3.

The in vivo efficacy of all 32 ionizable lipids was assessed. LNPs carrying firefly luciferase (Fluc)‐encoded mRNA were administered intravenously at a dose of 0.1 mg kg^−1^. The luminescence levels in the liver and spleen, and lung were measured 6 h after i.v. administration, and the intensity was found to vary dramatically, depending on the tail structure (Figure S3a–c, Supporting Information). Since the data indicated that our lipid library is promising for efficient mRNA delivery to the liver and spleen, we focused on those organs in this study. Data was visualized as a contour plot for symmetry and the total carbon number (**Figure** **5**a,b). These figures clearly indicate that highly symmetric lipids promote mRNA expression in the liver and spleen. Additionally, the mRNA was strongly expressed in the liver with total carbon numbers of C14–C18 and in the spleen with a total carbon number of C11–C14, suggesting that the total carbon number of the branched tail controls organ selectivity.

To understand the factors that determine the mRNA expression levels in each organ, 12 LNPs carrying Cy5‐mRNA were administered and their biodistribution was evaluated (Figure S3d,e, Supporting Information). In the branched lipid library, a 7500‐fold difference in luminescence intensity was observed in the liver, whereas the Cy5 fluorescence intensity in the liver only differed by twofold and there was no confirmed correlation (Figure 5c). This indicates that biodistribution alone cannot explain mRNA expression in the liver. For the spleen, a correlation between luminescence intensity and biodistribution was identified, except for CL4F 6–4 (Figure 5d). This indicated that biodistribution was an important factor for splenic mRNA expression.

To understand the significant gap between mRNA expression and distribution in the liver, we explored the impact of LNPs’ properties in relation to the experimental parameters on efficacy. It has been established that apparent pKa is an important parameter that is used to determine the in vivo fate of LNPs. For example, a pKa value of 6–7 is optimal for effective siRNA/mRNA delivery to the hepatocytes in the liver.^[^ ^9^, ^44^ ^]^ When plotting the measured pKa versus luminescence intensity in the liver for 32 LNPs, LNPs with an apparent pKa between 5.9 and 6.3 effectively induced mRNA expression in the branched lipid library (Figure 5e). The apparent pKa is thus a useful parameter by which to predict in vivo efficacy and is in agreement with previous knowledge. However, it alone cannot explain the mechanism, as a 10‐fold difference in luminescence is observed even if it was within the optimal pKa range.

Further analysis revealed that hemolytic activity in an acidic environment was also correlated with efficacy (Figure 5f), which is consistent with the knowledge that endosomal escape is a rate‐limiting step.^[^ ^45^, ^46^ ^]^ Exceptionally, CL4F 6–4 induces hemolysis at low concentrations despite its low efficacy in vivo. The cellular uptake of CL4F 6–4 LNPs was very low in vitro (Figure S4, Supporting Information), suggesting that they cannot introduce mRNA into the cytoplasm even if its hemolytic activity is higher.

We have thus proposed that the chemical “structure” of the branched tail determines the “properties” of the LNPs such as apparent pKa and ionization ability (hemolysis activity), which in turn affects their in vivo “function.” To elucidate if branched hydrophobic scaffolds regulate the properties and function of LNPs, two types of symmetric ionizable lipid (CL4F 6–4 and CL4F 14–12) were mixed in molar ratios ranging from 65:35 to 20:80. As expected, an increase in the ratio of CL4F 6–4 was attributed to a higher pKa value and increase in hemolytic activity due to increased molecular rotation and the protonatable space available (Figure 5f,g). When CL4F 6–4 and CL4F 14–12 were mixed at 50% each, the LNP properties approached optimal values and may have contributed to a 10‐fold higher efficient delivery than those alone (Figure 5g). The study shows that the properties of the LNPs can be precisely controlled by varying the mixing ratio of ionizable lipids with different tail structures to maximize their functions.

### 2.7 CRISPR/Cas9 Genome Editing Using Fully Symmetric Lipid CL4F 8–6

**Figure 6:**
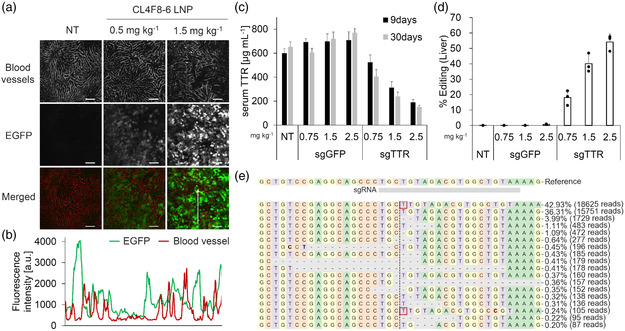
CL4F8‐6 LNPs enable CRISPR/Cas9 genome editing in vivo. a) Intrahepatic EGFP expression was observed following the administration of EGFP mRNA/CL4F8‐6 LNPs (0.5 or 1.5 mg kg^−1^ EGFP mRNA, 24 h). Representative images showed EGFP‐positive areas (green) and blood vessels visualized by DyLight649‐tomato lectin (red). Scale bars: 100 μm. b) Intensity profile of white allow indicated that EGFP was expressed mainly in hepatocytes. c) Serum TTR levels 9 and 30 d after administration was measured by ELISA. (0.75 or 1.5 or 2.5 mg kg^−1^ total RNA, Cas9 mRNA/sgRNA weight ratio was 1:2, *n* = 3) d) CL4F 8–6 LNPs carrying Cas9 mRNA and sgRNA were i.v. administrated into Balb/c mice. Mouse liver DNA sequencing was performed 30 d after administration and the genome editing outcomes were analyzed. *n* = 3. e) The distribution of the identified alleles around each cleavage site was visualized. Nucleotides are indicated by unique colors (A = green; C = red; G = yellow; and T = purple). Substitutions are shown in bold font. Red rectangles highlight the inserted sequences. Horizontal dashed lines indicate deleted sequences. The vertical dashed line indicates the predicted cleavage site.

CL4F 8–6 is a fully symmetric lipid that forms LNPs with favorable apparent pKa and membrane disruption abilities; CL4F 8–6 LNP achieved the most efficient Fluc‐mRNA expression in the liver among the branched lipid library. After 6 months of storage at 4 °C, the particle size and encapsulation efficiency of CL4F 8–6 LNPs were unchanged when compared to CL4F 14–2 LNPs (short, branched chain) (Figure S5a–c, Supporting Information), due to the restrictions in the rotational motion by the branched‐chain, as described above (Figure 3a). It is reported that LNPs targeted to the liver are adsorbed by protein coronas, mainly Apolipoprotein E (ApoE), in the blood and accumulate in liver hepatocytes via a low‐density lipoprotein receptor (LDLR).^[^ ^47^, ^48^ ^]^ To determine which cells the mRNA expressed, intrahepatic enhanced green fluorescent protein (EGFP) expression was observed following the administration of EGFP mRNA/CL4F8–6 LNPs. The microscopic observations revealed a clearly lower localization of EGFP fluorescence on blood vessels, so we consider that mRNA expression occurs mainly in hepatocytes in a dose‐dependent manner (**Figure** **6**a,b, and S6, Supporting Information). To investigate the applicability of our lipids for genome editing in hepatocytes, LNPs co‐encapsulated with Cas9 mRNA and single guide ribonucleic acid (RNA) against transthyretin (sgTTR) were prepared. Transthyretin (TTR) is a protein mainly produced by hepatocytes and secreted into the blood. TTR knockdown by siRNA is approved for ATTR amyloidosis treatment,^[^ ^49^ ^]^ and genome editing by CRISPR/Cas9 is also in clinical trials.^[^ ^18^ ^]^ CL4F 8–6 LNPs carrying Cas9 mRNA and sgTTR with different ratios from 2:1 to 1:2 were administered, with 1:2 being optimal for efficient TTR protein reduction (Figure S7, Supporting Information). To ensure the sequence specificity and eliminate the unexpected effects of lipids themselves, LNPs encapsulating sgTTR or sgGFP were compared in the same experiment (Table S4, Supporting Information). ELISA experiments showed a 77% reduction in serum TTR after 30 days of treatment with 2.5 mg kg^−1^ total RNA (mRNA/sgTTR = 1:2) (Figure 6c). In addition, mouse DNA sequencing was performed and the genome editing outcomes were analyzed. Genome editing efficiency was 54% in the liver at a single dose of 2.5 mg kg^−1^ (Figure 6d). sgTTR‐treated or sgGFP‐treated animals showed no editing in the spleen (Figure S8, Supporting Information). The distribution of alleles identified around the targeted locus is dominated by single nucleotide insertions, with the rest being deletions of a few bases (Figure 6e), consistent with the characteristic pattern of spCas9 reported in several studies.^[^ ^50^, ^51^ ^]^ These mutations lead to frameshifts and loss of function. These data illustrate the potential of CL4F 8–6 for long RNA delivery and genome editing.

## 3 Conclusion

Branched‐tail lipids are commonly used for the delivery of mRNA, however, the exact role of the branched chains remains unclear. To address this, we designed an α‐branched lipid library in which the length of each carbon chain can be easily controlled. The structural descriptions in the library used two simple parameters, symmetry, and total carbon number, which allowed for direct comparisons. We have demonstrated that highly symmetric lipids with a higher total carbon number form more viscous LNPs and can contribute to particle stabilization. In addition, we confirmed that branched chains maximize the ionization ability of headgroups and promote endosomal escape. Thus, chemical structure and LNP properties were found to be strongly linked and have an impact on activity. Specifically, branched lipids with a high level of symmetry contributed optimal properties for efficient intracellular delivery and stable formulations. For example, the symmetric CL4F 8–6 carried Cas9 mRNA and sgRNA simultaneously and induced 54% genome editing after a single dose. This data allows for deeper insights into branched tails and may guide novel lipid design in the future.

## 4 Experimental Section

4.1

4.1.1

##### Formulations of mRNA‐LNPs

The lipid molar ratio was fixed for the ionizable lipid : chol : DSPC : DMG‐mPEG2k at 50 : 38.5 : 10 : 1.5, respectively. The ratio of nitrogen to phosphate was fixed at 10. Each lipid was diluted in ethanol to a final concentration of 8 mm. The mRNA solution (46.1 μg mL^−1^) was prepared using citrate buffer (50 mm, pH 3.5). The lipid and mRNA solutions were mixed at 3 and 9 mL min^−1^ using the NanoAssemblr (Precision NanoSystems). After dilution with 20 mm HEPES buffer (9% sucrose, pH 7.45), the resulting LNP solutions were ultrafiltrated twice using Amicon Ultra‐15 (molecular weight cutoff (MWCO) 100 kDa, Millipore).

The size of the LNPs was measured using a Zetasizer Nano ZSP instrument (Malvern Instruments, Malvern, UK). The encapsulation efficiency and total concentration of the mRNAs were both measured using the Ribogreen assay, as described previously.^[^ ^52^ ^]^

##### Microviscosity Measurements

DCVJ (Sigma‐Aldrich, St. Louis, MO, USA) labeling of the LNPs was performed by adding 10 mm DCVJ dissolved in DMSO to the lipid solution at 0.1 mol% before formulation. The DCVJ‐labelled LNPs were empty and dialyzed against the D‐PBS(‐). For the fluorescence measurements, 200 μL of the DCVJ‐labelled LNPs (1.5 mm, 1.5 μm as DCVJ) were added to black 96‐well plates. The plates were incubated for 5 min at the set temperature (30, 40, 50, and 60 °C) and fluorescence was then measured using the EnSight Multimode Plate Reader (PerkinElmer, US). The samples were excited at 440 nm and the fluorescence spectra at 460–650 nm were obtained. To correct the DCVJ fluorescence intensity, DCVJ‐derived absorbance was also measured after solubilizing the particles. Then, 10% SDS (50 μL) was added to the LNP (450 μL) and the mixture was incubated at 60 °C for 10 min with gentle mixing at 700 rpm. Absorbance at 350–550 nm for the 320 μL samples was measured. To estimate the viscosity, R (fluorescence–absorption ratio) was calculated following the method of Chwastek et al.^[^ ^32^ ^]^ Specifically, the integral of the fluorescence spectrum at 460–660 nm was divided by the integral of the absorbance spectrum at 390–510 nm. The membrane microviscosity of each LNP was estimated based on a calibration curve prepared with a glycerol‐methanol mixture.^[^ ^53^ ^]^

##### Measurement of pKa and the Ionization Ability of the LNPs

The apparent pKa of the LNPs was determined as previously described.^[^ ^19^ ^]^ Briefly, TNS was diluted with each buffer and the pH ranged from 3.5–9.5 in 0.5 increments to a final concentration of 0.8 μm. The LNP was diluted with saline to 1 mm as the total lipid concentration. When improving the ionization ability, the LNPs were diluted with 0.1% or 1.0% Triton X‐100 to 1 mm and incubated at 25 °C for 10 min with gentle mixing at 700 rpm. In black 96‐well plates, 12 μL of LNP and 188 μL of TNS solutions for each pH were mixed. The fluorescence intensity was measured using an EnSight Multimode Plate Reader (PerkinElmer, US) with excitation at 321 nm and emission at 447 nm. The apparent pKa was calculated as the pH giving rise to the half‐maximal fluorescent intensity. The ionization ability was determined as the fluorescence intensity value at pH 5.0.

##### Animals

The experimental protocols were all reviewed and approved by the Hokkaido University Animal Care Committee in accordance with the guidelines for the care and use of laboratory animals. Balb/c mice (6–8 weeks old) were sourced from Japan SLC (Shizuoka, Japan). Mice were maintained on a regular 12 h light/12 h dark cycle in a specific animal facility at Hokkaido University. Five female Balb/c mice were housed in each cage. The mice were fed a pelleted mouse diet (cat# 5053, LabDiet, USA) and water ad libitum.

##### Hemolysis Assay

Fresh RBCs were collected from a Balb/c mouse and suspended in 20 mm DL‐ maleate buffer (pH 5.0, 130 mm NaCl). Step‐diluted LNPs (3.125–250 μm as a total lipid concentration) were mixed with an RBC suspension and incubated at 37 °C for 30 min with mixing at 900 rpm. After the removal of unhemolyzed RBCs by centrifugation (4 °C, 400g, 5 min), the absorbance of the supernatant at 545 nm was measured. The samples were incubated with 0.5 w v^−1^% Triton X‐100 as a positive control and those without LNPs were used as a negative control. The percentage of hemolysis (%hemolysis) was calculated as a percentage of the absorbance of the positive control. The LNP concentration for 50% hemolysis was calculated using a four‐parameter logistic curve fit analysis in Graphpad Prism 6.

##### Intrahepatic EGFP Expression

Balb/c mice (6–8 weeks old, female) were intravenously administered with the LNPs carrying CleanCap EGFP mRNA (5 moU) (TriLink BioTechnologies, US) at a dose of 0.5 or 1.5 mg kg^−1^. Then, 10 min before the collection of the liver tissues, the liver endothelial cells were stained with 20 μg per mouse of the Lycopersicon esculentum lectin, DyLight 649 Conjugate. Liver tissues were collected 24 h after the LNP treatment. Intrahepatic EGFP expression was observed using a Nikon A1 (Nikon Co. Ltd. Tokyo, Japan). Images were captured using 20× or 40× magnification.

##### In Vivo Fluc mRNA Delivery

Balb/c mice (6–8 weeks old, female) were intravenously administered with the LNPs carrying CleanCap FLuc mRNA (5 moU) (TriLink BioTechnologies, US) at a dose of 0.1 mg kg^−1^ for screening. Six hours after injection, the mice were injected with VivoGlo Luciferin In Vivo Grade (Promega, US) dissolved with PBS(−) via the tail vein at 1.5 mg per mouse. 3 min after injection, tissues were collected and imaged using an in vivo imaging system 200 series (Perkin Elmer, US).

##### In Vivo Biodistribution

Balb/c mice (6–8 weeks old, female) were intravenously administered with each of the LNPs carrying CleanCap Cyanine 5 FLuc mRNA (5 moU) (TriLink BioTechnologies, US) at a dose of 0.5 mg kg^−1^. Mouse tissues were isolated 3 h after each of the LNP treatments. The fluorescence intensity was measured using an in vivo imaging system 200 series (Perkin Elmer, US).

##### Mouse TTR Editing

CleanCap Cas9 mRNA (5 moU) (TriLink BioTechnologies, US) and sgTTR (Integrated DNA Technologies, US) were mixed to a weight ratio of 1:2 and simultaneously encapsulated in LNPs. Balb/c mice (6–8 weeks old, female) were intravenously administered with Cas9 mRNA/sgTTR LNPs at a dose of 0.75, 1.5, or 2.5 mg kg^−1^ total RNA. After 9 and 30 days of the LNP treatment, blood was collected from the mouse tail vein, then left at 25 °C for 1 h before being centrifuged (25 °C, 1000 g, 15 min) to collect the serum. Serum TTR protein was quantified using a Prealbumin ELISA Kit (Aviva Systems Biology Corp., US) according to the manufacturer's protocol. A four‐parameter logistic curve model was used to determine the TTR concentration. DNA was extracted and purified from the liver and spleen using NucleoSpin Tissue (Takara Bio, Japan). DNA was amplified using two‐step tailed PCR and sequencing was performed using an Illumina MiSeq instrument in paired‐end mode. The primers for the TTR locus were as follows: forward 5′‐TCG TCG GCA GCG TCA GAT GTG TAT AAG AGA CAG GCT TTG GAA ACA ATG CTG TCT AT‐3′ and reverse 5′‐GTC TCG TGG GCT CGG AGA TGT GTA TAA GAG ACA GTG GGC TTT CTA CAA GCT TAC C‐3′. The genome editing outcomes were analyzed using CRISPResso2.^[^ ^54^ ^]^

##### Data Analysis

The results are presented as the mean ± standard deviation (SD) unless otherwise mentioned. The physiochemical properties, microviscosity, and in vivo efficacy results were visualized using JMP software (ver. 14.0.0). The data was analyzed using an unpaired Student's *t*‐test or one‐way analysis of variance (ANOVA) followed by Dunnett's post‐tests for comparison of multiple groups using Graphpad Prism 6. Statistical significance was indicated by a *P* < 0.05.

## Conflict of Interest

K.H., Y.S., M.T., S.S., A.Ot., Y.M., T.S. and H.H. are authors on the patent WO2022/071 582 (A1) relating to ionizable lipid with branched scaffolds and methods of use thereof. K.H., M.T., S.S., A.Ot., Y.M., T.S., M.M. and A.Ok. were at the time of study employees of the Nitto Denko corporation.

## Author Contributions

K. H. and Y. S. contributed equally to this work. Conceptualization and methodology: K.H., Y.S., A.Ok., and H.H. Investigation, K.H., Y.S., M.T., S.S., A.Ot., Y.M., and T.S. Writing (original draft): K.H. Writing (review and editing): Y.S. and H.H. Supervision: Y.S., T.S., M.M., A.Ok., and H.H.

## Supporting information

Supplementary Material

## Data Availability

The data that support the findings of this study are available from the corresponding author upon reasonable request.
