# Branching Ionizable Lipids Can Enhance the Stability, Fusogenicity, and Functional Delivery of mRNA

**DOI:** 10.1002/smsc.202300285

**Published:** 2023-12-20

**Authors:** Kazuki Hashiba, Yusuke Sato, Masamitsu Taguchi, Sachiko Sakamoto, Ayaka Otsu, Yoshiki Maeda, Takuya Shishido, Masao Murakawa, Arimichi Okazaki, Hideyoshi Harashima

*Small Sci.* **2023**, *03*, 2200071

DOI: 10.1002/smsc.202200071

Reference [20] (W. He et al. *Mol. Ther.* **2014**, *22*, 359) in the published article is an incorrect citation. The reference is hereby corrected as:

[20] M. Cornebise, E. Narayanan, Y. Xia, E. Acosta, L. Ci, H. Koch, J. Milton, S. Sabnis, T. Salerno, K. E. Benenato, *Adv. Funct. Mater.* **2022**, *32*, 2106727.

The existing ref. [20] by He et al. is re‐cited in the article in the introduction as: “The headgroup is the positively charged moiety and often has a tertiary amine, of which there are a wide range of types, such as alkyl and cyclic amines.^[^^20^^]^”

