# Supplementary material for: Branching Ionizable Lipids Can Enhance the Stability, Fusogenicity, and Functional Delivery of mRNA
Source: Small Sci. 2022 Nov 9;3(1):2200071. doi: 10.1002/smsc.202200071 (PMC11935957; doi:10.1002/smsc.202200071)
Supplement: Supplementary file 1 — Supplementary Material [file SMSC-3-2200071-s001.pdf]

Supporting Information for

Branching ionizable lipids can enhance the stability, fusogenicity, and functional delivery of mRNA

Kazuki Hashiba, Yusuke Sato, Masamitsu Taguchi, Sachiko Sakamoto, Ayaka Otsu, Yoshiki Maeda, Takuya Shishido, Masao Murakawa, Arimichi Okazaki, Hideyoshi Harashima

Contents

Supporting figures 3

Supporting tables 11

Lipid synthesis 17

Reference 34

Supporting figures

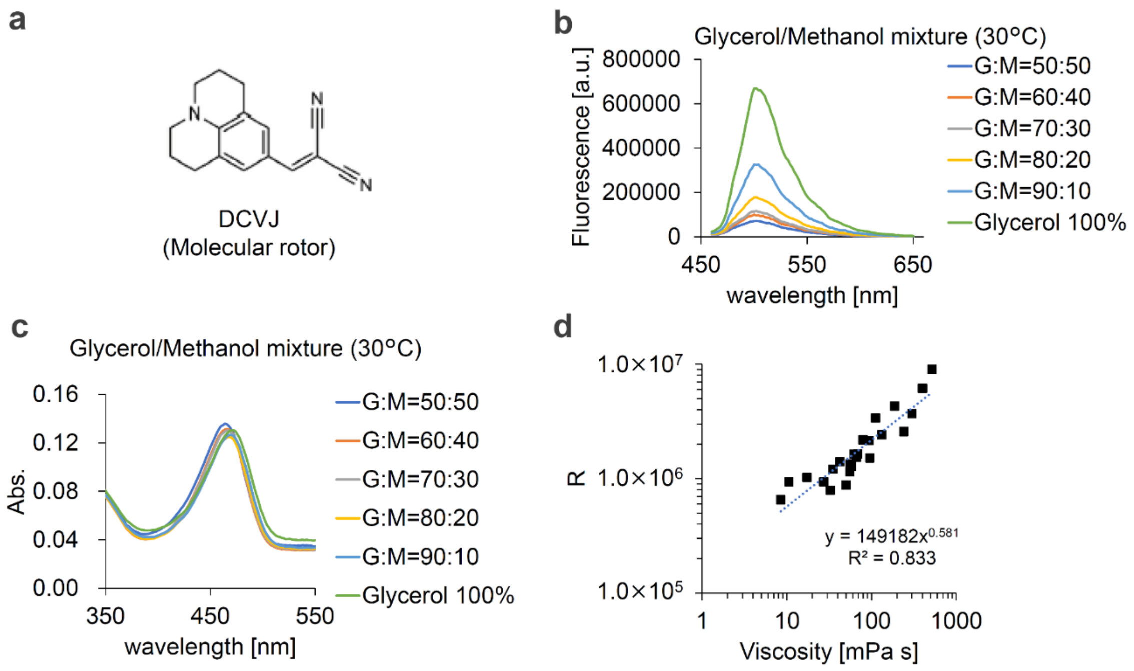

Figure S1. DCVJ responds to the microviscosity of the glycerol and methanol mixture.

a) Structure of DCVJ. b) DCVJ fluorescence responses to the microviscosity of different glycerol-methanol mixtures. c) DCVJ absorbance does not respond to the microviscosity in the different glycerol-methanol mixtures. d) Calibration curve between R (fluorescence-absorbance ratio) and viscosity. The viscosity values of the glycerol-methanol mixtures were referenced from Ghotli et al.[1]

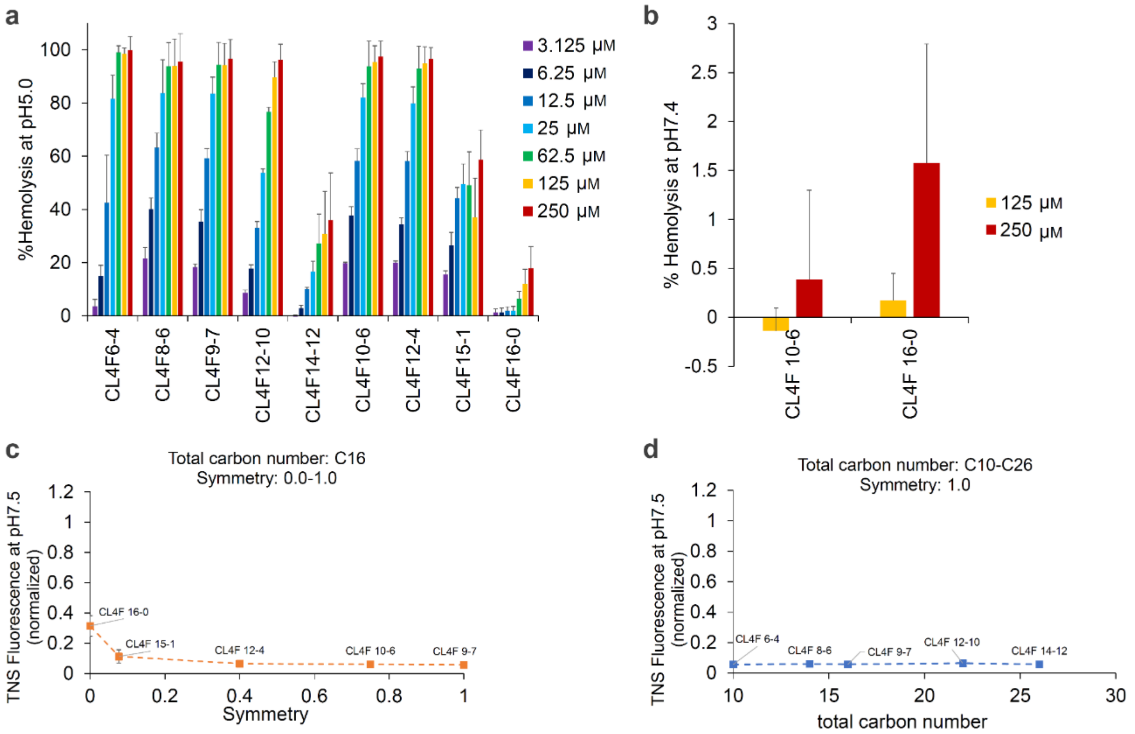

Figure S2. Tail structure impact on hemolysis efficiency at pH5.0 and hemolysis efficiency and ionization under physiological conditions are low.

a, b) Fresh red blood cells (RBCs) were suspended in 20 mM DL- maleate buffer (pH 5.0, 130 mM NaCl) or 10 mM HEPES/10 mM MES buffer (pH 7.4, 130 mM NaCl). Step-diluted LNPs (3.125–250 μM) were mixed with RBC suspensions and incubated at 37 °C for 30 min with mixing at 900 rpm. After the removal of the unhemolysed RBCs, the absorbance of the supernatant at 545 nm was measured. The %hemolysis was calculated as a percentage of the absorbance of the positive control. Data are represented as the mean ± SD, n = 3. c,d) The ionization at pH7.5 was measured by TNS assay. TNS fluorescence intensities were standardized using the same scale as in Figure 4b, c.

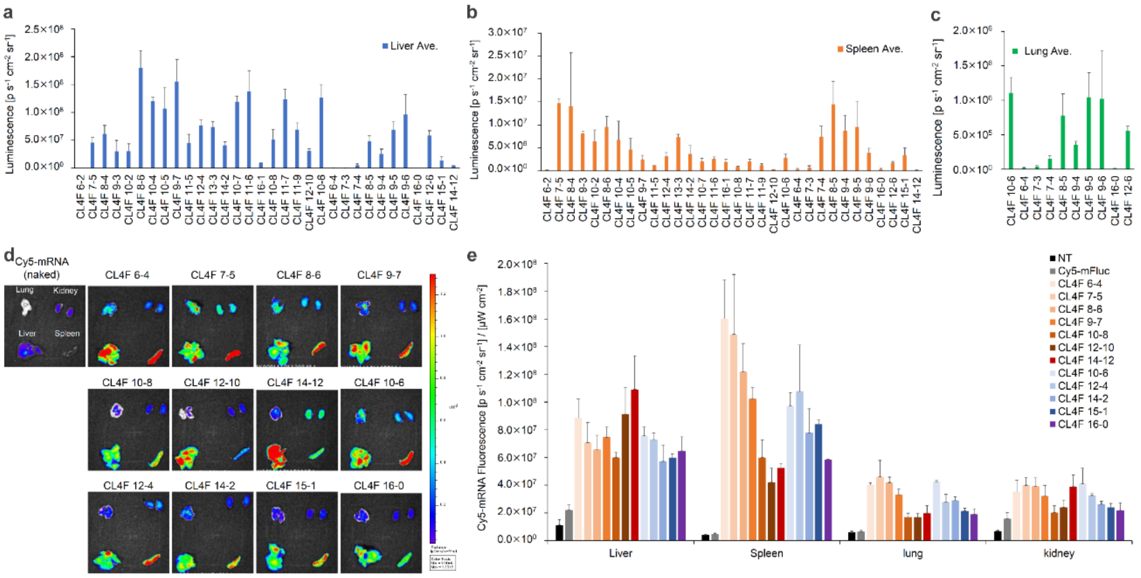

Figure S3. Fluc expression and Cy5-mRNA distribution after the i.v. administration of mRNA-LNPs.

a-c) Ex vivo bioluminescence in the liver, spleen, and lung were measured 6 h after the administration of 10-32 types of LNPs (0.1 mg kg^{-1} Fluc mRNA, n = 3) d) LNPs carrying Cy5-mRNA 0.5 mg kg^{-1} were administered via i.v. and the biodistribution evaluated 3 h after administration, n = 3. e) The region of interest (ROI) was specified to measure the average radiant efficiency.

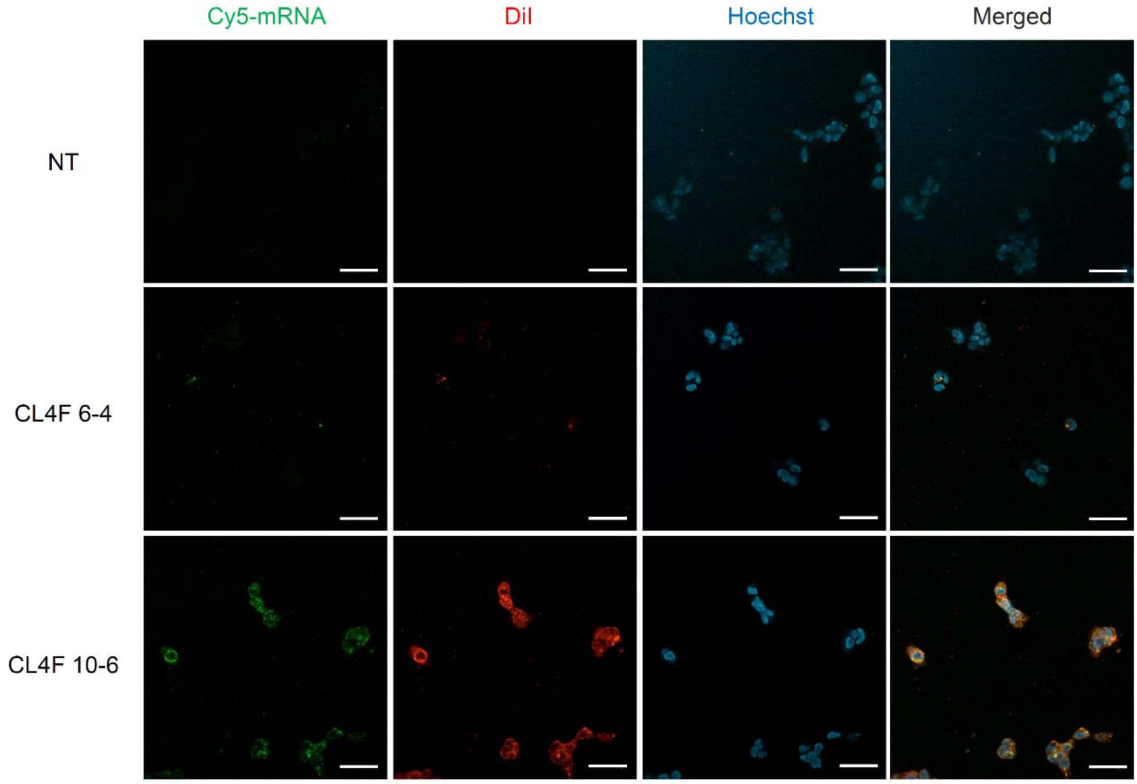

Figure S4. The cellular uptake of CL4F 6-4 LNPs was very low in vitro.

For in vitro transfection, DiI-labeled LNPs carrying CleanCap Cyanine 5 FLuc mRNA (5 moU) were added to glass bottom dishes preseeded with HEK293 cells at a dose of 0.5 µg/well. The cellular uptake of each LNP was observed using an LSM 900 (Zeiss, Germany) after a 3-hour incubation. Figure S4 represents Cy5-positive areas (green), DiI-positive areas (red), and nuclei, which were visualized by Hoechst33342 (blue). 50 µm Scale bars.

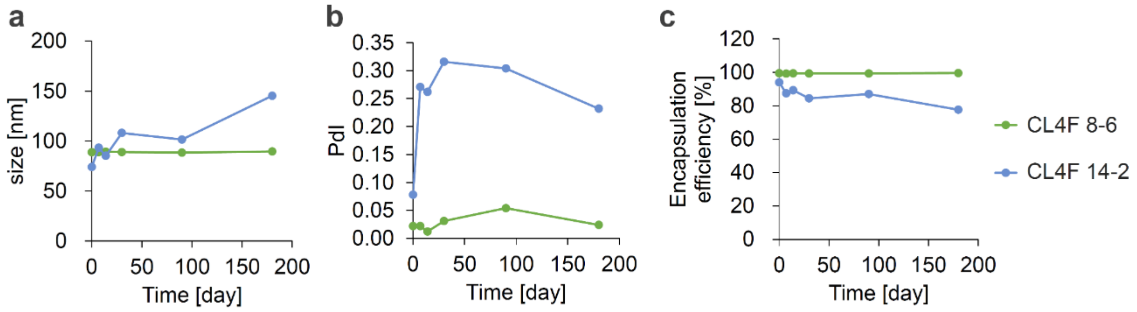

Figure S5. CL4F 8-6 LNPs are physically stable due to their longer branched side chains.

a-c) The physicochemical properties (average size, PDI value, and encapsulation efficiency) of CL4F 8-6 LNPs carrying Fluc mRNA did not change significantly over a 6-month period at 4°C.

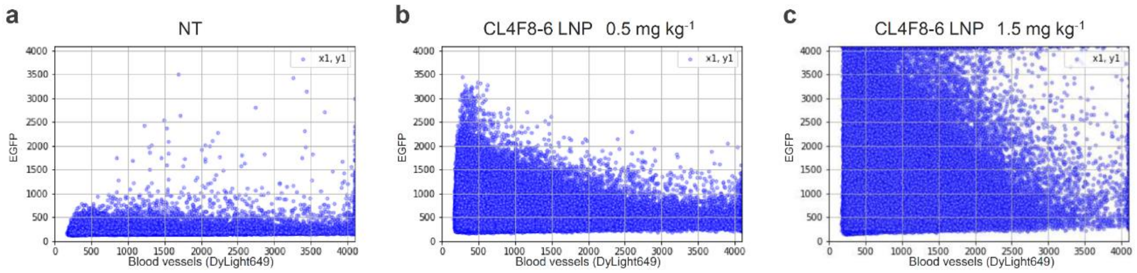

Figure S6. Scatterplot representing the localization of EGFP fluorescence on blood vessels.

Intrahepatic microscopic images after the administration of EGFP mRNA/CL4F8-6 LNPs (0.5 or 1.5 mg kg^{-1} EGFP mRNA, 24 h) were analyzed and the localization of EGFP fluorescence on blood vessels was visualized as a scatter plot. a) Non-treated. b) 0.5 mg kg^{-1} mRNA/CL4F8-6 LNPs. c) 1.5 mg kg^{-1} mRNA/CL4F8-6 LNPs. These data indicated that EGFP is mainly expressed in Dylight649 (blood vessels) low area.

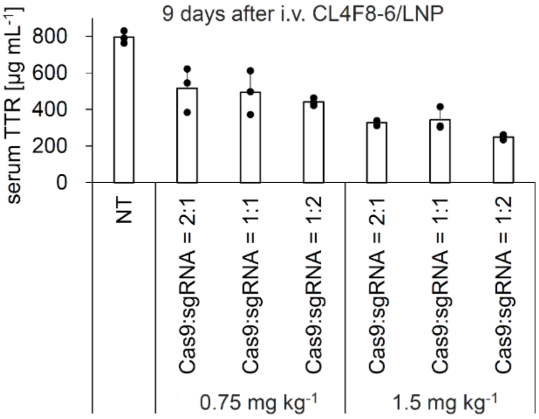

Figure S7. Influence of the Cas9 mRNA/sgTTR ratio on protein reduction.

Cas9 mRNA and sgTTR were administered at different ratios from 2:1 to 1:2. The results showed that the target protein tended to decrease when the ratio of Cas9 mRNA to sgTTR was 1:2.

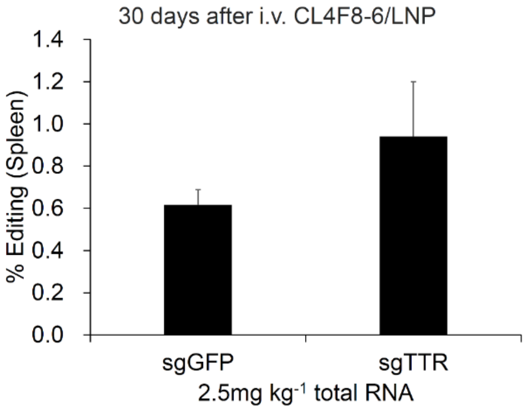

Figure S8. sgTTR-treated or sgGFP-treated animals showed no editing in the spleen. CL4F 8-6 LNPs carrying Cas9 mRNA and sgRNA were i.v. administered to Balb/c mice. Mouse spleen DNA sequencing was performed 30 d after administration and the genome editing outcomes were analyzed. n = 3.

Supporting tables

Table S1. ζ-Average, polydispersity index (PDI), mRNA encapsulation efficiency, and apparent pKa for all LNPs formulated in this study.

Lipid name	ζ-Average [nm]	PDI	% mRNA encapsulation	pKa
CL4F 6-2	218.20	0.069	41.4	6.40
CL4F 7-5	109.80	0.057	99.9	6.27
CL4F 8-4	101.60	0.020	99.7	6.27
CL4F 9-3	102.80	0.030	99.6	6.34
CL4F 10-2	93.60	0.068	98.6	6.35
CL4F 8-6	88.78	0.022	99.4	6.14
CL4F 10-4	84.92	0.041	99.8	6.19
CL4F 10-5	84.62	0.021	99.7	6.07
CL4F 9-7	81.30	0.004	99.2	6.05
CL4F 11-5	81.81	0.019	99.6	6.19
CL4F 12-4	84.57	0.015	99.0	6.16
CL4F 13-3	83.05	0.044	96.3	6.22
CL4F 14-2	74.02	0.078	94.0	6.29
CL4F 10-7	79.68	0.039	99.8	5.99
CL4F 11-6	84.50	0.020	99.8	6.02
CL4F 16-0	158.30	0.225	86.2	6.81
CL4F 16-1	69.08	0.045	65.9	6.40
CL4F 10-8	81.99	0.021	99.8	5.99
CL4F 11-7	79.45	0.021	99.7	5.98
CL4F 11-9	81.13	0.014	99.8	5.92
CL4F 12-10	75.85	0.019	99.6	5.85
CL4F 10-6	82.66	0.006	99.6	6.05
CL4F 6-4	141.10	0.023	97.2	6.38
CL4F 7-3	133.90	0.025	98.1	6.39
CL4F 7-4	113.50	0.032	98.8	6.33
CL4F 8-5	94.65	0.043	99.1	6.23
CL4F 9-4	95.29	0.002	99.1	6.26
CL4F 9-5	89.41	0.048	99.0	6.16

CL4F 9-6	89.18	0.021	99.1	6.10
CL4F 12-6	82.84	0.017	99.7	6.03
CL4F 15-1	106.4	0.143	88.9	6.32
CL4F 14-12	97.41	0.043	97.9	5.83

Table S2. Result of multiple regression analysis based on the result of factor analysis.

	Coefficient	SE	t-value	p-value
Intercept	-0.222	17.992	-0.012	0.99031
total carbon number	6.763	1.034	6.542	0.00002
symmetry	38.993	9.845	3.961	0.00163

Table S3. Physicochemical properties for each LNP were assessed after being stored at each given temperature (4°C, 25°C, or 40°C).

			4°C			25°C		40°C		
	Day	0	14	30	90	7	30	3	7	30
CL4F 6-2	Size [nm]	218.2	227.3	233.7	231.4	227.3	232.1	234.9	232.7	222.0
	PDI	0.07	0.10	0.09	0.12	0.06	0.11	0.11	0.11	0.20
	EE [%]	41.4	68.0	65.6	68.0	66.3	66.9	64.9	71.0	62.3
CL4F 7-5	Size [nm]	109.8	113.7	133.5	116.5	107.5	116.8	118.9	127.9	206.1
	PDI	0.06	0.03	0.02	0.05	0.04	0.002	0.04	0.06	0.11
	EE [%]	99.9	99.6	99.6	99.7	99.4	98.9	93.5	83.1	29.2
CL4F 8-4	Size [nm]	101.6	103.3	102.7	105.2	102.3	106.3	106.3	107.7	205.4
	PDI	0.02	0.03	0.02	0.04	0.02	0.03	0.02	0.05	0.13
	EE [%]	99.7	99.6	99.6	99.4	99.0	97.7	95.9	87.6	52.2
CL4F 9-3	Size [nm]	102.8	109.2	109.4	112.7	104.6	112.1	138.3	162.8	226.3
	PDI	0.03	0.02	0.02	0.03	0.02	0.01	0.12	0.13	0.10
	EE [%]	99.6	99.4	99.4	99.1	99.3	98.4	82.3	65.6	17.6
CL4F 10-2	Size [nm]	93.6	98.6	103.1	107.1	99.1	109.7	171.5	209.7	278.2
	PDI	0.07	0.08	0.06	0.08	0.15	0.22	0.17	0.16	0.18
	EE [%]	98.6	98.1	97.8	98.2	97.6	96.9	53.7	39.3	6.8
CL4F 8-6	Size [nm]	88.8	89.4	89.0	88.4	88.7	91.8	88.1	92.4	126.2
	PDI	0.02	0.01	0.03	0.05	0.03	0.02	0.02	0.007	0.15
	EE [%]	99.4	99.4	99.3	99.3	99.3	98.7	98.2	98.4	76.6
CL4F 10-4	Size [nm]	84.9	85.9	85.2	85.8	84.6	89.1	106.5	114.1	152.3
	PDI	0.04	0.04	0.05	0.03	0.05	0.03	0.05	0.04	0.14
	EE [%]	99.8	99.3	99.1	99.2	99.1	98.8	95.8	93.8	73.7
CL4F 10-5	Size [nm]	84.6	86.3	85.1	84.3	97.3	101.9	89.9	89.7	98.9
	PDI	0.02	0.02	0.04	0.02	0.10	0.12	0.03	0.07	0.07
	EE [%]	99.7	99.4	99.3	99.4	92.5	92.2	97.8	97.2	93.7
CL4F 9-7	Size [nm]	81.3	82.1	82.2	81.3	86.1	89.6	83.7	82.1	82.9
	PDI	0.004	0.06	0.05	0.02	0.06	0.05	0.05	0.02	0.05
	EE [%]	99.2	98.9	98.9	99.1	97.4	97.1	98.9	98.4	98.7
CL4F 11-5	Size [nm]	81.8	81.1	81.1	78.1	81.1	82.4	85.5	92.4	129.4
	PDI	0.02	0.03	0.03	0.02	0.04	0.03	0.07	0.12	0.21
	EE [%]	99.6	99.3	99.3	99.3	99.6	99.4	99.4	99.2	97.0
CL4F 12-4	Size [nm]	84.5	86.6	85.3	84.3	87.1	93.7	101.8	101.1	164.3
	PDI	0.02	0.08	0.09	0.05	0.05	0.08	0.06	0.07	0.25
	EE [%]	99.0	98.6	98.5	98.6	98.0	96.7	94.3	94.3	55.4
CL4F 13-3	Size [nm]	83.0	90.8	98.9	95.4	126.0	155.4	113.5	110.8	152.1
	PDI	0.04	0.19	0.24	0.23	0.26	0.19	0.20	0.12	0.49
	EE [%]	96.3	92.9	90.9	91.1	79.0	67.6	81.3	80.8	75.8
CL4F 14-2	Size [nm]	74.0	85.2	108.1	101.5	130.7	169.1	115.6	120.5	164.5
	PDI	0.08	0.26	0.31	0.30	0.40	0.35	0.20	0.17	0.19
	EE [%]	94.0	89.3	84.4	87.0	74.7	64.2	75.2	75.1	57.6
CL4F 10-7	Size [nm]	79.6	80.3	78.3	78.7	84.6	95.9	81.8	80.9	82.9
	PDI	0.04	0.01	0.03	0.004	0.06	0.11	0.03	0.02	0.02
	EE [%]	99.8	99.8	99.8	99.9	98.4	95.5	99.7	99.5	99.3
CL4F 11-6	Size [nm]	84.5	83.2	82.1	80.4	82.5	88.0	86.8	88.3	90.3
	PDI	0.02	0.05	0.02	0.02	0.04	0.01	0.03	0.02	0.04
	EE [%]	99.8	99.8	99.9	100.0	99.8	99.1	99.6	99.6	99.3
CL4F 16-1	Size [nm]	69.0	98.2	96.1	107.7	98.0	98.7	1281	1962	5110
	PDI	0.05	0.14	0.16	0.17	0.18	0.20	0.39	0.05	1.00
	EE [%]	65.9	63.3	63.9	67.7	60.9	57.2	58.0	51.1	53.5
CL4F 10-8	Size [nm]	82.0	82.1	82.6	81.7	82.0	83.4	84.6	84.1	86.0
	PDI	0.02	0.03	0.003	0.01	0.02	0.03	0.03	0.003	0.05
	EE [%]	99.8	99.8	99.4	100.0	99.9	99.8	99.8	99.9	99.7
CL4F 11-7	Size [nm]	79.4	79.9	79.9	78.4	81.1	82.7	86.7	85.9	87.8
	PDI	0.02	0.04	0.06	0.01	0.06	0.07	0.06	0.08	0.06
	EE [%]	99.7	99.5	99.4	99.8	99.6	99.5	99.5	99.5	99.4
CL4F 11-9	Size [nm]	81.1	80.3	79.3	80.3	82.1	83.0	82.2	82.6	82.0

	PDI	0.01	0.04	0.03	0.006	0.01	0.07	0.01	0.02	0.04
	EE [%]	99.8	99.8	99.8	99.9	99.8	99.5	99.8	99.7	99.6
CL4F 12-10	Size [nm]	75.8	77.1	76.8	76.5	79.9	82.7	85.8	84.7	82.8
	PDI	0.02	0.06	0.03	0.07	0.06	0.08	0.05	0.06	0.07
	EE [%]	99.6	99.5	99.4	99.5	99.5	99.2	99.5	99.4	99.5
CL4F 10-6	Size [nm]	82.6	84.0	81.6	82.1	88.1	98.7	85.0	84.3	87.0
	PDI	0.01	0.02	0.003	0.03	0.04	0.08	0.01	0.02	0.07
	EE [%]	99.6	99.6	99.6	99.8	97.3	93.8	98.7	98.5	98.1
CL4F 6-4	Size [nm]	141.1	149.7	150.5	153.5	150.4	147.9	153.9	163.7	170.6
	PDI	0.02	0.05	0.008	0.05	0.02	0.05	0.06	0.09	0.18
	EE [%]	97.2	98.4	99.0	98.6	98.4	99.0	94.2	70.9	58.9
CL4F 7-3	Size [nm]	133.9	145.5	146.8	156.1	147.5	149.1	150.3	165.3	178.5
	PDI	0.03	0.07	0.03	0.06	0.06	0.07	0.05	0.10	0.11
	EE [%]	98.1	98.6	99.1	98.4	98.4	98.8	78.9	60.9	52.4
CL4F 7-4	Size [nm]	113.5	121.3	125.3	138.5	119.6	128.2	118.6	125.6	194.1
	PDI	0.03	0.04	0.06	0.05	0.05	0.04	0.05	0.06	0.10
	EE [%]	98.8	99.0	99.2	99.2	97.7	97.7	96.7	93.6	69.4
CL4F 8-5	Size [nm]	94.6	96.4	93.5	99.6	94.3	97.8	96.1	97.1	96.4
	PDI	0.04	0.03	0.05	0.02	0.04	0.05	0.02	0.05	0.03
	EE [%]	99.1	99.3	99.2	99.5	98.7	98.4	99.1	98.5	98.3
CL4F 9-4	Size [nm]	95.2	95.9	94.8	98.9	96.2	98.7	96.3	99.1	131.7
	PDI	0.002	0.04	0.01	0.04	0.03	0.04	0.02	0.06	0.12
	EE [%]	99.1	99.2	99.1	99.5	98.7	98.7	97.7	97.2	88.3
CL4F 9-5	Size [nm]	89.4	88.8	87.8	91.3	90.6	98.0	89.5	90.5	88.0
	PDI	0.05	0.06	0.04	0.03	0.03	0.08	0.02	0.01	0.01
	EE [%]	99.0	99.2	99.1	99.6	98.0	97.1	98.6	98.7	98.8
CL4F 9-6	Size [nm]	89.1	88.9	85.4	91.0	87.5	88.7	88.7	90.3	87.3
	PDI	0.02	0.03	0.01	0.02	0.02	0.08	0.02	0.004	0.01
	EE [%]	99.1	99.4	99.0	99.7	99.0	98.9	98.9	99.1	99.2
CL4F 16-0	Size [nm]	158.3	160.6	157.3	250.3	163.8	178.1	191.9	200.8	243.3
	PDI	0.23	0.25	0.29	0.41	0.21	0.32	0.12	0.13	0.17
	EE [%]	86.2	91.4	85.1	96.0	93.9	93.9	86.3	84.7	90.8
CL4F 12-6	Size [nm]	82.8	82.8	81.7	81.9	86.3	94.8	82.3	83.6	82.0
	PDI	0.01	0.04	0.05	0.02	0.06	0.08	0.04	0.03	0.03
	EE [%]	99.7	99.5	98.9	99.9	97.8	98.0	99.0	99.0	99.1
CL4F 15-1	Size [nm]	106.4	113.2	117.5	125.4	113.9	122.2	152.2	174.4	476.6
	PDI	0.14	0.13	0.15	0.15	0.15	0.13	0.34	0.33	0.56
	EE [%]	88.9	85.5	82.0	70.3	87.2	87.9	66.3	45.7	11.0
CL4F 14-12	Size [nm]	97.4	101	104	105	96.5	99.6	99.9	96.8	98.5
	PDI	0.04	0.02	0.03	0.05	0.03	0.03	0.02	0.01	0.01
	EE [%]	97.8	98.6	97.1	99.4	99.4	97.9	98.6	99.5	98.6

Table S4. Guide RNA target sequences.

sgTTR	5′-UUACAGCCACGUCUACAGCA-3′
sgGFP	5′-CUCGUGACCACCCUGACCUA-3′

Lipid synthesis

General information

All simple chemicals were purchased from Tokyo Chemical Industry (Tokyo, Japan) or FUJIFILM Wako Pure Chemical Corporation (Osaka, Japan) and were used without further purification. ^{1}H NMR spectra were obtained from a JEOL ECZ400 or ECP400 instrument (Tokyo, Japan) and chemical shifts were expressed in parts per million (ppm) with respect to the residual solvent peak. The following abbreviations were used to express the multiplications: s = singlet; d = doublet; t = triplet; and m = multiplet. All reactions were monitored using thin-layer chromatography on pre-coated thin-layer chromatography (TLC) plates (Millipore), stained with a bromocresol green solution or p-anisaldehyde solution. The products were purified using a Biotage Selekt automated chromatography system. All compounds were provided as a mixture of all possible stereoisomers.

General Procedure A (Branched fatty acid synthesis from dimethyl malonate)

NaH (7.56 mmol) was suspended in tetrahydrofuran (THF) (18 mL) and stirred at 0 °C for 10 min. Dimethyl malonate (7.56 mmol) was then added, and the mixture was again stirred at 0 °C for another 10 min. Two different iodoalkanes are used to synthesize one fatty acid. The first iodoalkane (7.56 mmol) was then added and allowed to react overnight at 25 °C. Additional NaH (11.34 mmol) was added to the reaction mixture and the mixture was stirred at 0 °C for 10 min. The second iodoalkane (11.34 mmol) was then added and allowed to react overnight at 25 °C. After quenching with acetic acid, the mixture was washed with brine. After evaporation, the residue was dissolved in ethanol (16 mL) and hydrolyzed using an 8N NaOH solution (5 mL). The reaction was carried out overnight at 60 °C. After quenching with the 6N hydrochloric acid, the mixture was washed with brine. The organic phase was dried over Na_2SO_4. After the removal of the solvent, the crude was heated at 160 °C for 2 h for decarboxylation. The residue was purified by flash chromatography [ODS, H2O (10 mM ammonium acetate: acetonitrile/isopropanol, 50:50)] to obtain the branched fatty acid as a colorless oil.

General Procedure B (Branched fatty acid synthesis from linear fatty acids)

Linear fatty acids (10.28 mmol) were dissolved in THF (36 mL), followed by 1.0 M lithium diisopropylamide in THF/hexanes (24 mmol) which was added dropwise at -20°C and the mixture was stirred at 0°C for 30 min. N,N'-Dimethylpropyleneurea (DMPU) (18 mL) was then added and the mixture was stirred at 0 °C for 60 min. Iodoalkane (23.2 mmol) was then added, and the reaction was carried out overnight at 10 °C. After

quenching with 2N hydrochloric acid, the reaction mixture was diluted with diethyl ether and washed with brine. After removal of the solvent in vacuo, the residue was purified by flash chromatography [ODS, (10 mM ammonium acetate: acetonitrile/isopropanol, 50:50)] to obtain the branched fatty acids as a colorless oil.

General Procedure C (esterification to yield ionizable lipid)
7-(4-dipropylamino)butyl)tridecane-1,7,13-triol was synthesized as previously described [A17]. Fatty acids (2.4 mmol) were added to 7-(4-dipropylamino)butyl)tridecane-1,7,13-triol (387.7 mg, 1.0 mmol) in anhydrous DCM (5 mL). Dimethylaminopyridine (DMAP) (12.2 mg, 0.1 mmol) and EDCI-HCl (576 mg, 3.0 mmol) were added to the mixture and the reaction mixture was stirred at 25 °C overnight. After evaporation of the solvent, the residue was suspended in EtOAc and washed with a 0.5N NaOH solution, and then brine. The organic phase was dried over Na_2SO_4. Evaporation of the solvent produced crude as a yellow oily residue. The residue was purified using flash chromatography [ODS, H_2O (0.1% trifluoroacetic acid)/acetonitrile:isopropanol = 50:50 (0.1% TFA)] and (SiO_2, DCM/MeOH). Ionizable lipids were then produced as a colorless oil.

Branched fatty acids synthesized by General Procedure A:

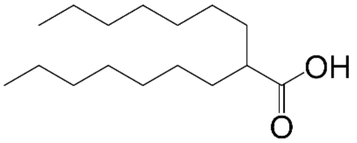

2-Heptylnonanoic acid (9-7): The final product obtained was a colorless oil (840 mg, 98%). ^{1}H NMR (400 MHz, $CDCl_3$, ppm) δ0.87 (m, 6H), 1.20–1.35 (m, 24H), 1.45 (m, 2H), 1.60 (m, 2H), and 2.32 (m, 1H).

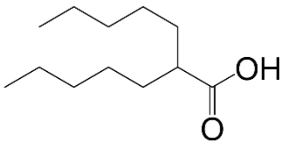

2-Pentylheptanoic acid (7-5): The final product obtained was a colorless oil (450 mg, 60%). ^{1}H NMR (400 MHz, $CDCl_3$, ppm) δ0.87 (m, 6H), 1.20–1.35 (m, 12H), 1.45 (m, 2H), 1.60 (m, 2H), and 2.32 (m, 1H).

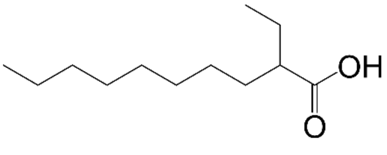

2-Ethyldecanoic acid (10-2): The final product obtained was a colorless oil (544 mg, 30%). ^{1}H NMR (400 MHz, $CDCl_3$, ppm) δ0.87 (m, 6H), 1.18–1.35 (m, 12H), 1.45 (m, 2H), 1.60 (m, 2H), and 2.32 (m, 1H).

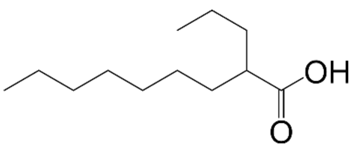

2-Propylnonanoic acid (9-3): The final product obtained was a colorless oil (960 mg, 63%). ^{1}H NMR (400 MHz, $CDCl_3$, ppm) δ0.89 (m, 6H), 1.20–1.35 (m, 12H), 1.45 (m, 2H), 1.60 (m, 2H), and 2.33 (m, 1H).

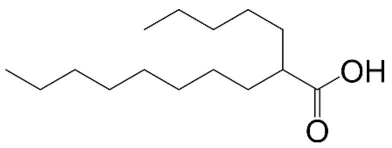

2-Pentyldecanoic acid (10-5): The final product obtained was a colorless oil (1600 mg, 66%). ^{1}H NMR (400 MHz, $CDCl_3$, ppm) δ0.87 (m, 6H), 1.20–1.35 (m, 22H), 1.45 (m, 2H), 1.60 (m, 2H), and 2.32 (m, 1H).

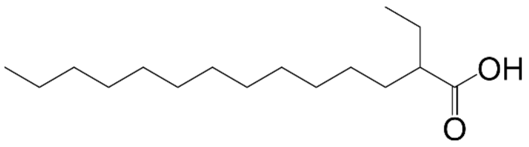

2-Ethyltetradecanoic acid (14-2): The final product obtained was a colorless oil (1160 mg, 63%). ^{1}H NMR (400 MHz, $CDCl_3$, ppm) δ0.87 (m, 6H), 1.20–1.35 (m, 24H), 1.45 (m, 2H), 1.60 (m, 2H), and 2.25 (m, 1H).

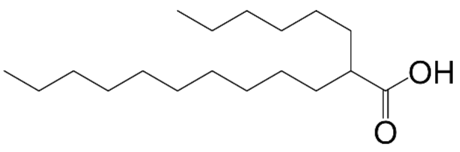

2-Hexyldodecanoic acid (12-6) : The final product obtained was a colorless oil (550 mg, 26%). ^{1}H NMR (400 MHz, $CDCl_3$, ppm) δ0.87 (m, 6H), 1.20–1.35 (m, 28H), 1.45 (m, 2H), 1.60 (m, 2H), and 2.33 (m, 1H).

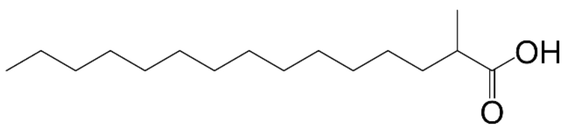

2-Metylpentadecanoic acid (15-1): The final product obtained was a colorless oil (1400

mg, 55%). ^{1}H NMR (400 MHz, $CDCl_3$, ppm) δ0.87 (t, 3H), 1.17 (d, 3H), 1.20–1.35 (m, 26H), 1.44 (m, 1H), 1.66 (m, 1H), and 2.44 (m, 1H).

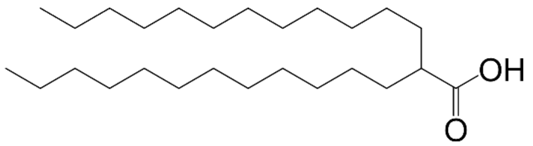

2-Dodecyltetradecanoic acid (14-12): The final product obtained was a colorless oil (3400 mg, 86%). ^{1}H NMR (400 MHz, $CDCl_3$, ppm) δ0.87 (m, 6H), 1.19–1.35 (m, 44H), 1.47 (m, 2H), 1.60 (m, 2H), and 2.32 (m, 1H).

Branched fatty acids synthesized by General Procedure B:

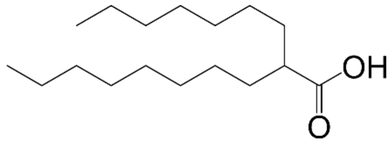

2-Heptyldecanoic acid (10-7): The final product obtained was a colorless oil (400 mg, 29%). ^{1}H NMR (400 MHz, $CDCl_3$, ppm) δ0.87 (m, 6H), 1.20–1.35 (m, 26H), 1.45 (m, 2H), 1.60 (m, 2H), and 2.32 (m, 1H).

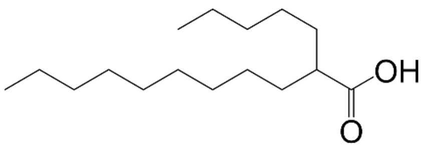

2-Pentylundecanoic acid (11-5): The final product obtained was a colorless oil (470 mg, 18%). ^{1}H NMR (400 MHz, $CDCl_3$, ppm) δ0.87 (m, 6H), 1.20–1.35 (m, 24H), 1.45 (m, 2H), 1.60 (m, 2H), and 2.32 (m, 1H).

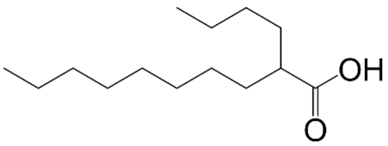

2-Butyldecanoic acid (10-4): The final product obtained was a colorless oil (900 mg, 38%). ^{1}H NMR (400 MHz, $CDCl_3$, ppm) δ0.87 (m, 6H), 1.20–1.35 (m, 20H), 1.45 (m, 2H), 1.60 (m, 2H), and 2.31 (m, 1H).

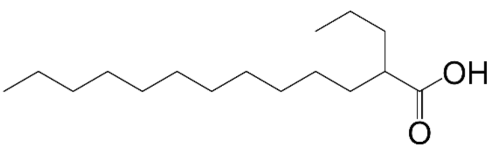

2-Propyltridecanoic acid (13-3): The final product was obtained was a colorless oil (1300

mg, 49%). ^{1}H NMR (400 MHz, $CDCl_3$, ppm) δ0.87 (m, 6H), 1.18–1.35 (m, 24H), 1.45 (m, 2H), 1.60 (m, 2H), and 2.34 (m, 1H).

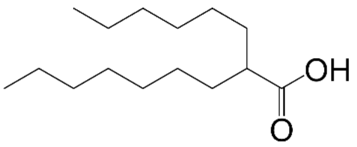

2-Hexylnonanoic acid (9-6): The final product obtained was a colorless oil (390 mg, 16%). ^{1}H NMR (400 MHz, $CDCl_3$, ppm) δ0.85 (m, 6H), 1.20–1.35 (m, 22H), 1.45 (m, 2H), 1.60 (m, 2H), and 2.32 (m, 1H).

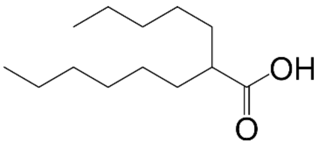

2-Pentyloctanoic acid (8-5): The final product obtained was a colorless oil (480 mg, 22%). ^{1}H NMR (400 MHz, $CDCl_3$, ppm) δ0.87 (m, 6H), 1.20–1.35 (m, 18H), 1.47 (m, 2H), 1.60 (m, 2H), 2.32 (m, 1H).

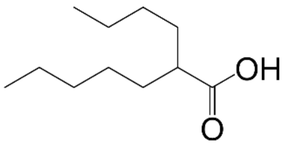

2-Butylheptanoic acid (7-4): The final product obtained was a colorless oil (1300 mg, 67%). ^{1}H NMR (400 MHz, $CDCl_3$, ppm) δ0.87 (m, 6H), 1.20–1.35 (m, 14H), 1.45 (m, 2H), 1.60 (m, 2H), and 2.34 (m, 1H).

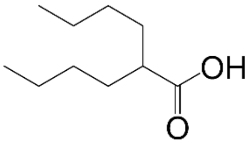

2-Butylhexanoic acid (6-4): The final product obtained was a colorless oil (1190 mg, 46%). ^{1}H NMR (400 MHz, $CDCl_3$, ppm) δ0.87 (m, 6H), 1.25–1.32 (m, 12H), 1.50 (m, 2H), 1.62 (m, 2H), and 2.33 (m, 1H).

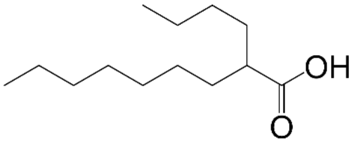

2-Butylnonanoic acid (9-4): The final product obtained was a colorless oil (1160 mg, 35%). ^{1}H NMR (400 MHz, $CDCl_3$, ppm) δ0.86 (m, 6H), 1.20–1.35 (m, 18H), 1.49 (m,

2H), 1.61 (m, 2H), and 2.34 (m, 1H).

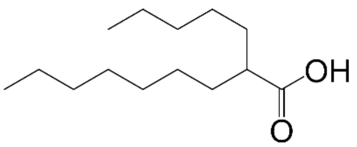

2-Pentylnonanoic acid (9-5): The final product obtained was a colorless oil (860 mg, 24%). ^{1}H NMR (400 MHz, $CDCl_3$, ppm) δ0.87 (m, 6H), 1.20–.35 (m, 20H), 1.48 (m, 2H), 1.61 (m, 2H), and 2.34 (m, 1H).

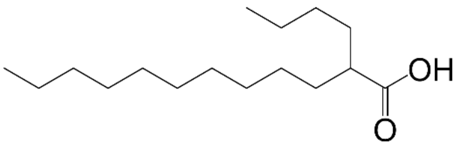

2-Butyldodecanoic acid (12-4): The final product obtained was a colorless oil (840 mg, 22%). ^{1}H NMR (400 MHz, $CDCl_3$, ppm) δ0.88 (m, 6H), 1.20–1.35 (m, 24H), 1.48 (m, 2H), 1.61 (m, 2H), and 2.34 (m, 1H).

Ionizable lipids synthesized by General Procedure C;

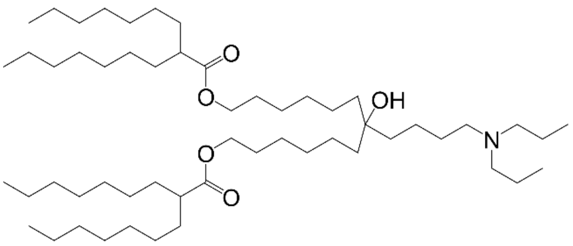

7-(4-(dipropylamino)butyl)-7-hydroxytridecane-1,13-diyl bis(2-heptylnonanoate) (CL4F 9-7): The final product obtained was a colorless oil (260 mg, 46.3%). ^{1}H NMR (400 MHz, $CDCl_3$, ppm) δ0.87 (m, 18H), 1.17–1.67 (m, 78H), 2.25–2.42 (m, 8H), and 4.06 (t, 4H). The m/z was 864.4 and the $[M+H]^+$ m/z was 865.

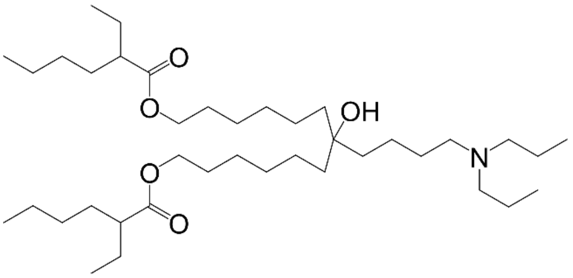

7-(4-(dipropylamino)butyl)-7-hydroxytridecane-1,13-diyl bis(2-ethylnohexanoate) (CL4F 6-2): The final product obtained was a colorless oil (175 mg, 27.4%). ^{1}H NMR

(400 MHz, $CDCl_3$, ppm) δ0.85 (m, 18H), 1.17–1.75 (m, 46H), 2.20–2.42 (m, 8H), and 4.06 (t, 4H). The m/z was 640.0 and the $[M+H]^+$ m/z was 641.

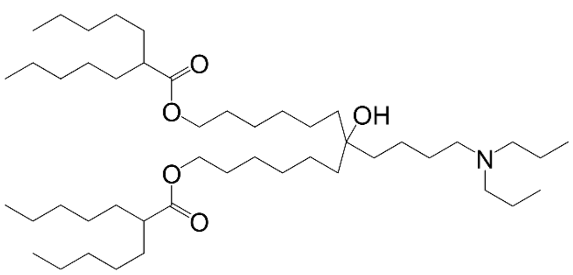

7-(4-(dipropylamino)butyl)-7-hydroxytridecane-1,13-diyl bis(2-pentylheptanoate) (CL4F 7-5): The final product obtained was a colorless oil (470 mg, 65.7%). ^1H NMR (400 MHz, $CDCl_3$, ppm) δ0.87 (m, 18H), 1.17–1.65 (m, 62H), 2.25–2.42 (m, 8H), and 4.04 (t, 4H). The m/z was 752.2 and the $[M+H]^+$ m/z was 753.

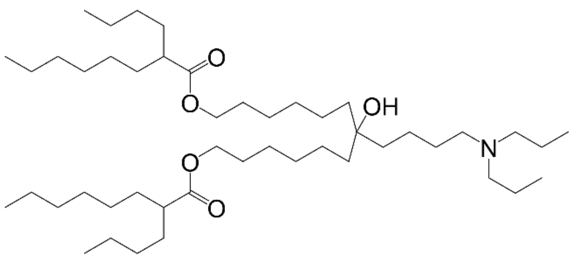

7-(4-(dipropylamino)butyl)-7-hydroxytridecane-1,13-diyl bis(2-butyloctanoate) (CL4F 8-4): The final product obtained was a colorless oil (568 mg, 88.8%). ^1H NMR (400 MHz, $CDCl_3$, ppm) δ0.87 (m, 18H), 1.17–1.67 (m, 62H), 2.25–2.42 (m, 8H), and 4.03 (t, 4H). The m/z was 752.2 and the $[M+H]^+$ m/z was 753.

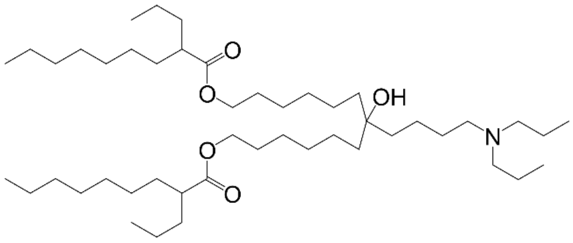

7-(4-(dipropylamino)butyl)-7-hydroxytridecane-1,13-diyl bis(2-propylnonanoate) (CL4F 9-3): The final product obtained was a colorless oil (320 mg, 42.6 %). ^1H NMR (400 MHz, $CDCl_3$, ppm) δ0.87 (m, 18H), 1.17–1.67 (m, 62H), 2.25–2.41 (m, 8H), and 4.04 (t, 4H). The m/z was 752.2 and the $[M+H]^+$ m/z was 753.

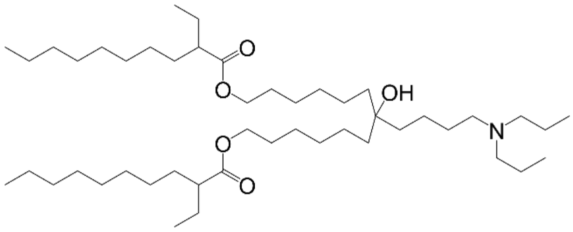

7-(4-(dipropylamino)butyl)-7-hydroxytridecane-1,13-diyl bis(2-ethyldecanoate) (CL4F 10-2): The final product obtained was a colorless oil (600 mg, 71.9%). ^{1}H NMR (400 MHz, $CDCl_3$, ppm) δ0.85 (m, 18H), 1.17–1.67 (m, 62H), 2.19–2.42 (m, 8H), and 4.06 (t, 4H). The m/z was 752.2 and the $[M+H]^+$ m/z was 753.

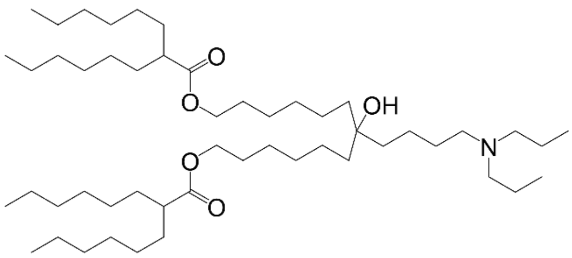

7-(4-(dipropylamino)butyl)-7-hydroxytridecane-1,13-diyl bis(2-hexyloctanoate) (CL4F 8-6): The final product obtained was a colorless oil (519 mg, 64.2%). ^{1}H NMR (400 MHz, $CDCl_3$, ppm) δ0.87 (m, 18H), 1.16–1.67 (m, 70H), 2.25–2.42 (m, 8H), and 4.02 (t, 4H). The m/z was 808.3 and the $[M+H]^+$ m/z was 809.

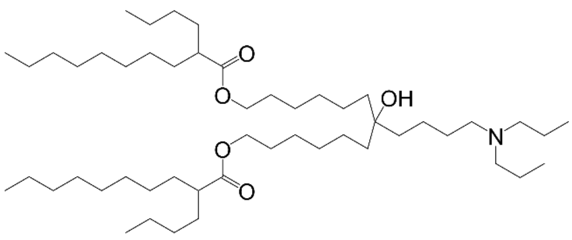

7-(4-(dipropylamino)butyl)-7-hydroxytridecane-1,13-diyl bis(2-butyldecanoate) (CL4F 10-4): The final product obtained was a colorless oil (450 mg, 55.7%). ^{1}H NMR (400 MHz, $CDCl_3$, ppm) δ0.87 (m, 18H), 1.15–1.67 (m, 70H), 2.25–2.45 (m, 8H), and 4.05 (t, 4H). The m/z was 808.3 and the $[M+H]^+$ m/z was 809.

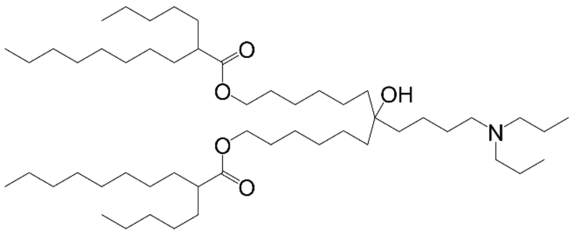

7-(4-(dipropylamino)butyl)-7-hydroxytridecane-1,13-diyl bis(2-pentyldenanoate) (CL4F 10-5): The final product obtained was a colorless oil (360 mg, 43.0%). ^{1}H NMR (400 MHz, $CDCl_3$, ppm) δ0.87 (m, 18H), 1.17–1.67 (m, 74H), 2.25–2.42 (m, 8H), and 4.03 (t, 4H). The m/z was 836.4 and the $[M+H]^+$ m/z was 837.

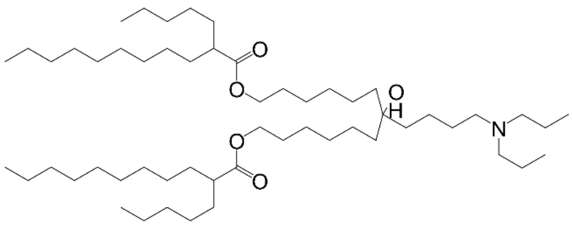

7-(4-(dipropylamino)butyl)-7-hydroxytridecane-1,13-diyl bis(2-pentylundecanoate) (CL4F 11-5): The final product obtained was a colorless oil (350 mg, 52.9%). ^{1}H NMR (400 MHz, $CDCl_3$, ppm) δ0.87 (m, 18H), 1.17–1.65 (m, 78H), 2.23–2.42 (m, 8H), and 4.04 (t, 4H). The m/z was 864.4 and the $[M+H]^+$ m/z was 865.

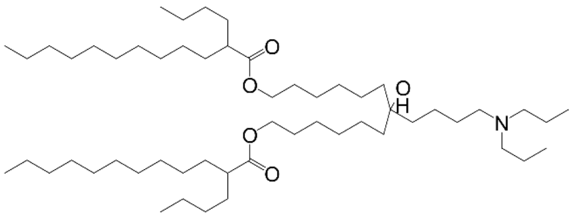

7-(4-(dipropylamino)butyl)-7-hydroxytridecane-1,13-diyl bis(2-butyldodecanoate) (CL4F 12-4): The final product obtained was a colorless oil (290 mg, 55.8%). ^{1}H NMR (400 MHz, $CDCl_3$, ppm) δ0.85 (m, 18H), 1.17–1.65 (m, 78H), 2.25–2.42 (m, 8H), and 4.04 (t, 4H). The m/z was 864.4 and the $[M+H]^+$ m/z was 865.

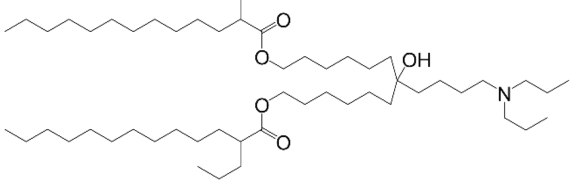

7-(4-(dipropylamino)butyl)-7-hydroxytridecane-1,13-diyl bis(2-propyltridecanoate) (CL4F 13-3): The final product obtained was a colorless oil (580 mg, 67.0%). ^{1}H NMR (400 MHz, $CDCl_3$, ppm) δ0.87 (m, 18H), 1.17–1.67 (m, 78H), 2.25–2.42 (m, 8H), and 4.03 (t, 4H). The m/z was 864.4 and the $[M+H]^+$ m/z was 865.

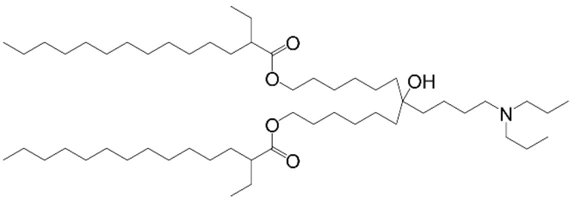

7-(4-(dipropylamino)butyl)-7-hydroxytridecane-1,13-diyl bis(2-ethyltetradecanoate) (CL4F 14-2): The final product obtained was a colorless oil (326 mg, 44.8%). ^{1}H NMR (400 MHz, $CDCl_3$, ppm) δ0.87 (m, 18H), 1.17–1.65 (m, 78H), 2.23 (m, 2H), 2.30–2.42 (m, 6H), and 4.04 (t, 4H). The m/z was 864.4 and the $[M+H]^+$ m/z was 865.

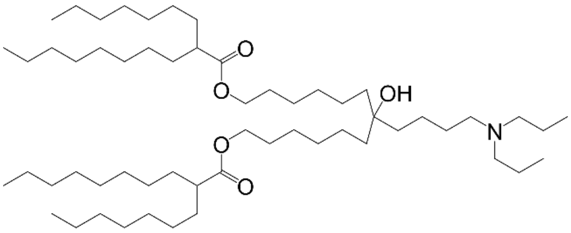

7-(4-(dipropylamino)butyl)-7-hydroxytridecane-1,13-diyl bis(2-heptyldecanoate) (CL4F 10-7): The final product obtained was a colorless oil (230 mg, 47.8%). ^{1}H NMR (400 MHz, $CDCl_3$, ppm) δ0.87 (m, 18H), 1.17–1.70 (m, 82H), 2.25–2.42 (m, 8H), and 4.03 (t, 4H). The m/z was 892.5 and the $[M+H]^+$ m/z was 893.

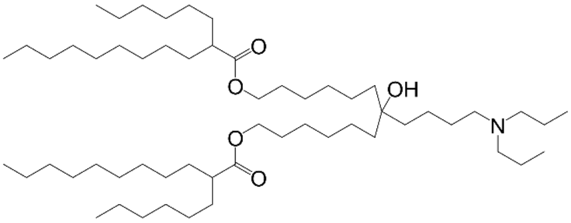

7-(4-(dipropylamino)butyl)-7-hydroxytridecane-1,13-diyl bis(2-hexylundecanoate) (CL4F 11-6): The final product obtained was a colorless oil (400 mg, 44.8%). ^{1}H NMR (400 MHz, $CDCl_3$, ppm) δ0.87 (m, 18H), 1.18–1.67 (m, 82H), 2.25–2.42 (m, 8H), 4.04 (t, 4H). The m/z was 892.5 and the $[M+H]^+$ m/z was 893.

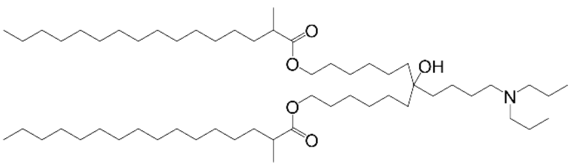

7-(4-(dipropylamino)butyl)-7-hydroxytridecane-1,13-diyl bis(2-methylhexadecanoate) (CL4F 16-1): The final product obtained was a colorless oil (606 mg, 67.9%). ^{1}H NMR

(400 MHz, $CDCl_3$, ppm) δ0.87 (m, 12H), 1.12 (d, 6H), 1.17–1.67 (m, 82H), 2.25–2.42 (m, 8H), and 4.02 (t, 4H). The m/z was 892.5 and the $[M+H]^+$ m/z was 893.

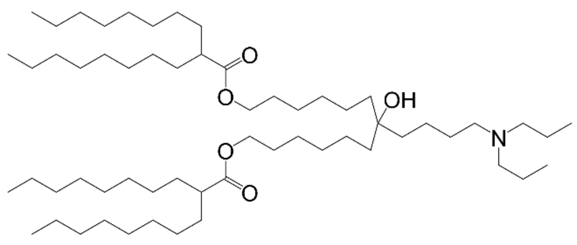

7-(4-(dipropylamino)butyl)-7-hydroxytridecane-1,13-diyl bis(2-octyldecanoate) (CL4F 10-8): The final product obtained was a colorless oil (260 mg, 43.5%). ^{1}H NMR (400 MHz, $CDCl_3$, ppm) δ0.87 (m, 18H), 1.17–1.67 (m, 86H), 2.23–2.45 (m, 8H), and 4.04 (t, 4H). The m/z was 920.5 and the $[M+H]^+$ m/z was 921.

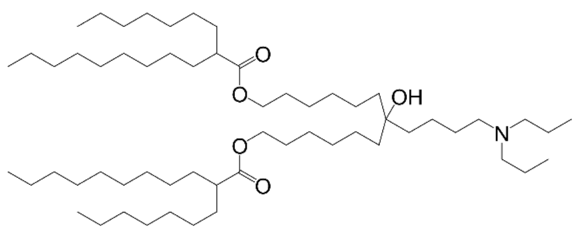

7-(4-(dipropylamino)butyl)-7-hydroxytridecane-1,13-diyl bis(2-heptylundecanoate) (CL4F 11-7): The final product obtained was a colorless oil (mg, %). ^{1}H NMR (400 MHz, $CDCl_3$, ppm) δ0.87 (m, 18H), 1.17–1.67 (m, 86H), 2.25–2.42 (m, 8H), and 4.06 (t, 4H). The m/z was 920.5 and the $[M+H]^+$ m/z was 921.

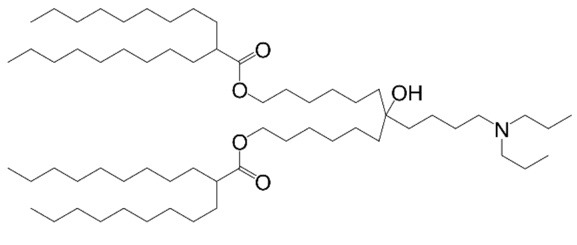

7-(4-(dipropylamino)butyl)-7-hydroxytridecane-1,13-diyl bis(2-nonylundecanoate) (CL4F 11-9): The final productobtained was a colorless oil (680 mg, 69.7%). ^{1}H NMR (400 MHz, $CDCl_3$, ppm) δ0.87 (m, 18H), 1.17–1.67 (m, 94H), 2.25–2.42 (m, 8H), and 4.05 (t, 4H). The m/z was 976.6 and the $[M+H]^+$ m/z was 977.

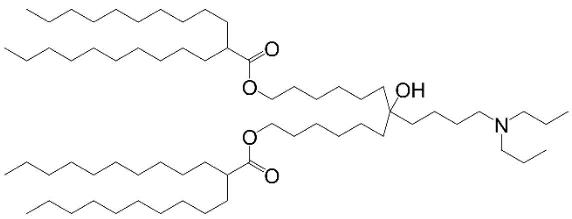

7-(4-(dipropylamino)butyl)-7-hydroxytridecane-1,13-diyl bis(2-decyldodecanoate) (CL4F 12-10): The final product obtained was a colorless oil (mg, %). ^{1}H NMR (400 MHz, $CDCl_3$, ppm) δ0.87 (m, 18H), 1.17–1.67 (m, 102H), 2.25–2.42 (m, 8H), 4.06 (t, 4H). The m/z was 1032.7 and the $[M+H]^+$ m/z was 1033.

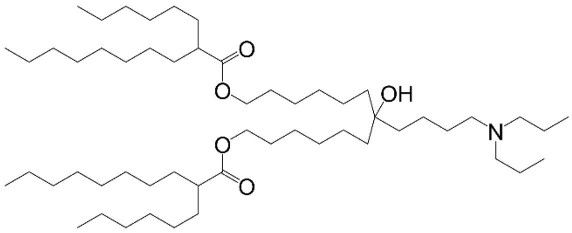

7-(4-(dipropylamino)butyl)-7-hydroxytridecane-1,13-diyl bis(2-hexyldecanoate) (CL4F 10-6): The final product obtained was a colorless oil (mg, %). ^{1}H NMR (400 MHz, $CDCl_3$, ppm) δ0.87 (m, 18H), 1.17–1.67 (m, 78H), 2.25–2.42 (m, 8H), and 4.06 (t, 4H). The m/z was 864.4 and the $[M+H]^+$ m/z was 865.

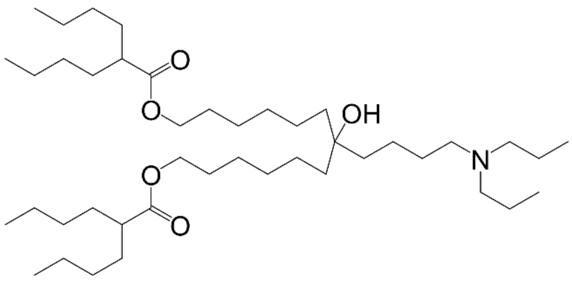

7-(4-(dipropylamino)butyl)-7-hydroxytridecane-1,13-diyl bis(2-butylhexanoate) (CL4F 6-4): The final product obtained was a colorless oil (360 mg, 53.5%). ^{1}H NMR (400 MHz, $CDCl_3$, ppm) δ0.87 (m, 18H), 1.17–1.67 (m, 54H), 2.25–2.41 (m, 8H), and 4.06 (t, 4H). The m/z was 696.1 and the $[M+H]^+$ m/z was 697.

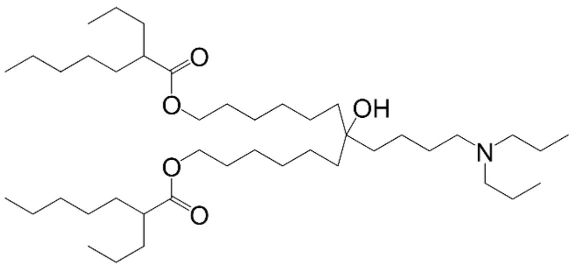

7-(4-(dipropylamino)butyl)-7-hydroxytridecane-1,13-diyl bis(2-propylheptanoate) (CL4F 7-3): The final product obtained was a colorless oil (465 mg, 66.9%). ^{1}H NMR (400 MHz, $CDCl_3$, ppm) δ0.88 (m, 18H), 1.17–1.67 (m, 54H), 2.27–2.47 (m, 8H), and 4.05 (t, 4H). The m/z was 696.1 and the $[M+H]^+$ m/z was 697.

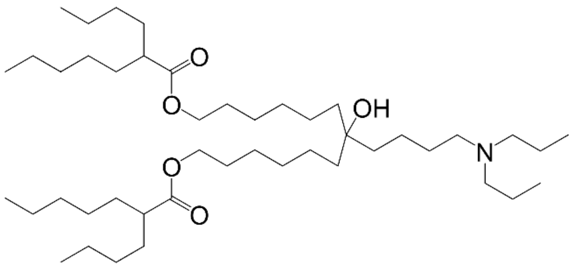

7-(4-(dipropylamino)butyl)-7-hydroxytridecane-1,13-diyl bis(2-butylheptanoate) (CL4F 7-4): The final product obtained was a colorless oil (550 mg, 76.0%). ^{1}H NMR (400 MHz, $CDCl_3$, ppm) δ0.87 (m, 18H), 1.17–1.65 (m, 58H), 2.25–2.42 (m, 8H), and 4.05 (t, 4H). The m/z was 724.2 and the $[M+H]^+$ m/z was 725.

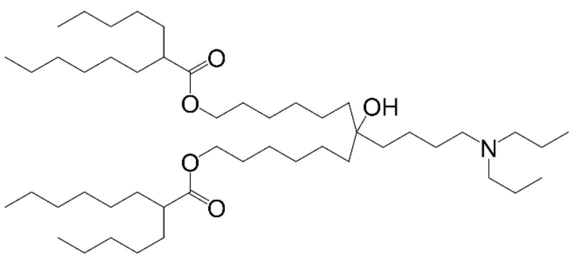

7-(4-(dipropylamino)butyl)-7-hydroxytridecane-1,13-diyl bis(2-pentyloctanoate) (CL4F 8-5): The final product obtained was a colorless oil (361 mg, 49.5%). ^{1}H NMR (400 MHz, $CDCl_3$, ppm) δ0.87 (m, 18H), 1.17–1.67 (m, 66H), 2.25–2.44 (m, 8H), 4.05 (t, 4H). The m/z was 780.3 and the $[M+H]^+$ m/z was 781.

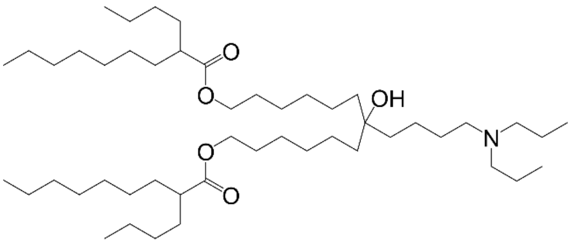

7-(4-(dipropylamino)butyl)-7-hydroxytridecane-1,13-diyl bis(2-butylnonanoate) (CL4F 9-4): The final product obtained was a colorless oil (510 mg, 65.4%). ^{1}H NMR (400 MHz, $CDCl_3$, ppm) δ0.87 (m, 18H), 1.15–1.67 (m, 66H), 2.25–2.47 (m, 8H), and 4.05 (t, 4H). The m/z was 780.3 and the $[M+H]^+$ m/z was 781.

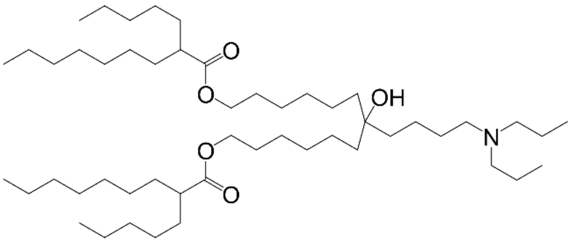

7-(4-(dipropylamino)butyl)-7-hydroxytridecane-1,13-diyl bis(2-pentylnonanoate) (CL4F 9-5): The final product obtained was a colorless oil (362 mg, 60.3%). ^{1}H NMR (400 MHz, $CDCl_3$, ppm) δ0.85 (m, 18H), 1.15–1.67 (m, 70H), 2.25–2.50 (m, 8H), and 4.05 (t, 4H). The m/z was 808.3 and the $[M+H]^+$ m/z was 809.

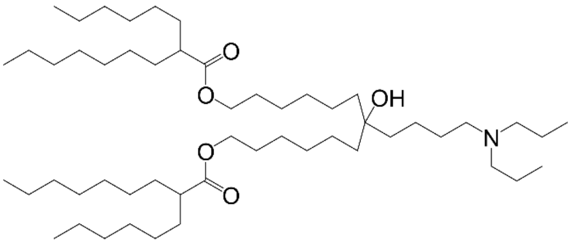

7-(4-(dipropylamino)butyl)-7-hydroxytridecane-1,13-diyl bis(2-hexylnonanoate) (CL4F 9-6): The final product obtained was a colorless oil (400 mg, 70.1%). ^{1}H NMR (400 MHz, $CDCl_3$, ppm) δ0.87 (m, 18H), 1.17–1.67 (m, 74H), 2.25–2.48 (m, 8H), and 4.05 (t, 4H). The m/z was 836.4 and the $[M+H]^+$ m/z was 937.

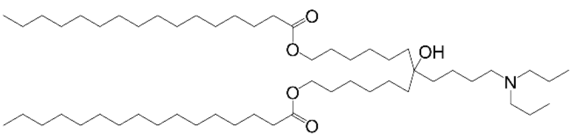

7-(4-(dipropylamino)butyl)-7-hydroxytridecane-1,13-diyl dipalmitate (CL4F 16-0): The final product obtained was a colorless oil (514 mg, 59.5%). ^{1}H NMR (400 MHz, $CDCl_3$, ppm) δ0.87 (m, 12H), 1.17–1.65 (m, 84H), 2.25 (m, 4H), 2.32–2.52 (m, 4H), and 4.04 (t,

4H). The m/z was 864.4 and the $[M+H]^+$ m/z was 865.

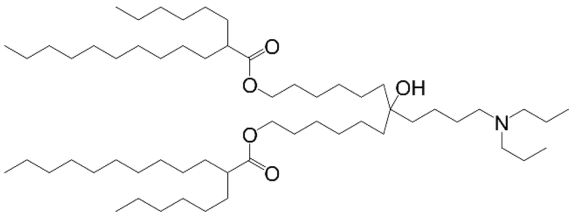

7-(4-(dipropylamino)butyl)-7-hydroxytridecane-1,13-diyl bis(2-hexyldodecanoate) (CL4F 12-6): The final product obtained was a colorless oil (400 mg, 65.9%). ^{1}H NMR (400 MHz, $CDCl_3$, ppm) δ0.87 (m, 18H), 1.17–1.67 (m, 86H), 2.25–2.47 (m, 8H), and 4.06 (t, 4H). The m/z was 920.5 and the $[M+H]^+$ m/z was 921.

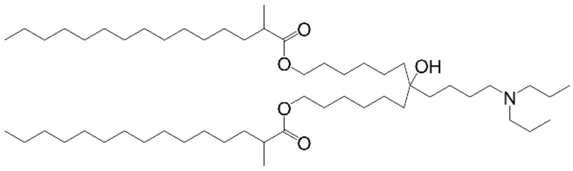

7-(4-(dipropylamino)butyl)-7-hydroxytridecane-1,13-diyl bis(2-metylpentadecanoate) (CL4F 15-1): The final product obtained was a colorless oil (380 mg, 60.1%). ^{1}H NMR (400 MHz, $CDCl_3$, ppm) δ0.78–0.99 (m, 12H), 1.12 (d, 6H), 1.17–1.67 (m, 78H), 2.35–2.82 (m, 8H), and 4.06 (t, 4H). The m/z was 864.4 and the $[M+H]^+$ m/z was 865.

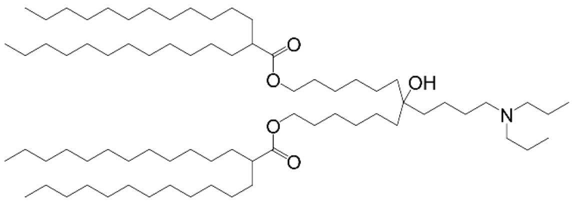

7-(4-(dipropylamino)butyl)-7-hydroxytridecane-1,13-diyl bis(2-dodecyltetradecanoate) (CL4F 14-12): The final product obtained was a colorless oil (750 mg, 65.5%). ^{1}H NMR (400 MHz, $CDCl_3$, ppm) δ0.87 (m, 18H), 1.17–1.69 (m, 118H), 2.25–2.70 (m, 8H), and 4.04 (t, 4H). The m/z was 1145.0 and the $[M+H]^+$ m/z was 1146.

Representative ^{1}H NMR spectra of branched fatty acids (7-5)

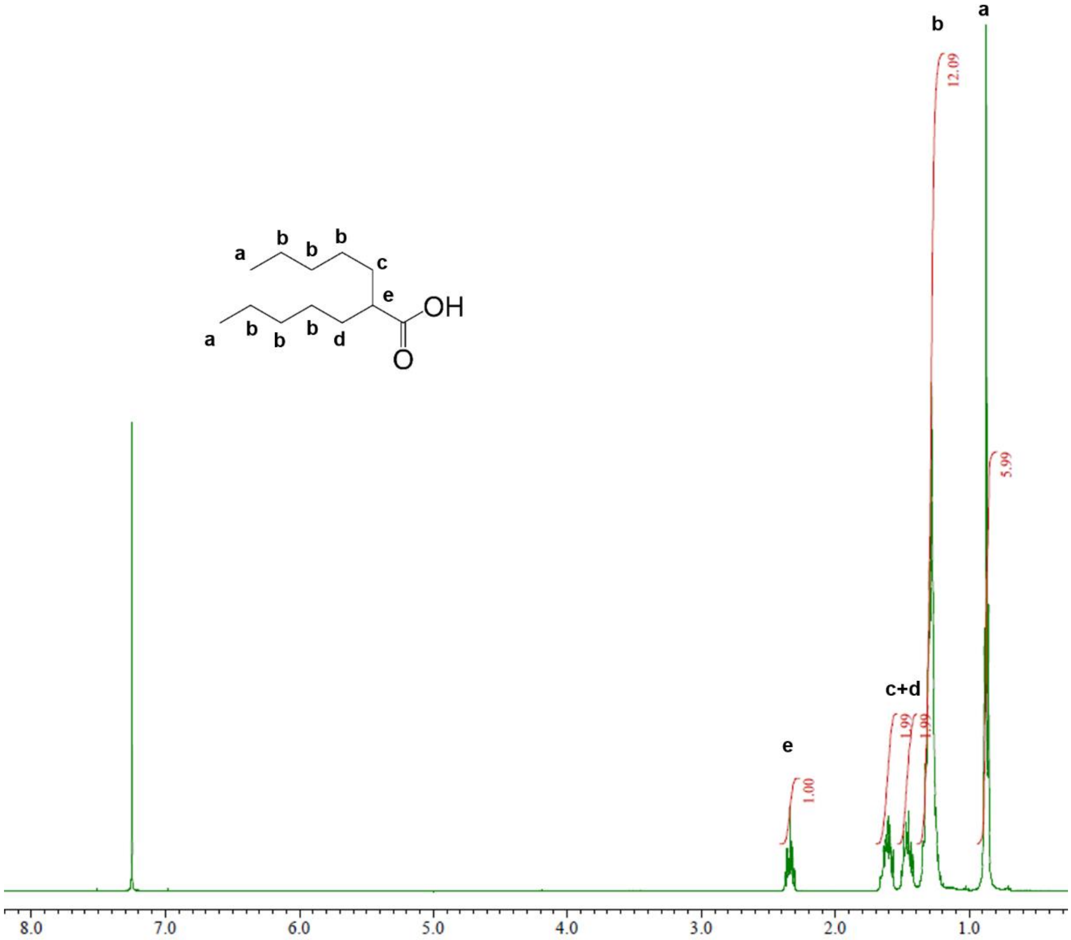

Representative ^{1}H NMR spectra for the ionizable lipid (CL4F 7-5)

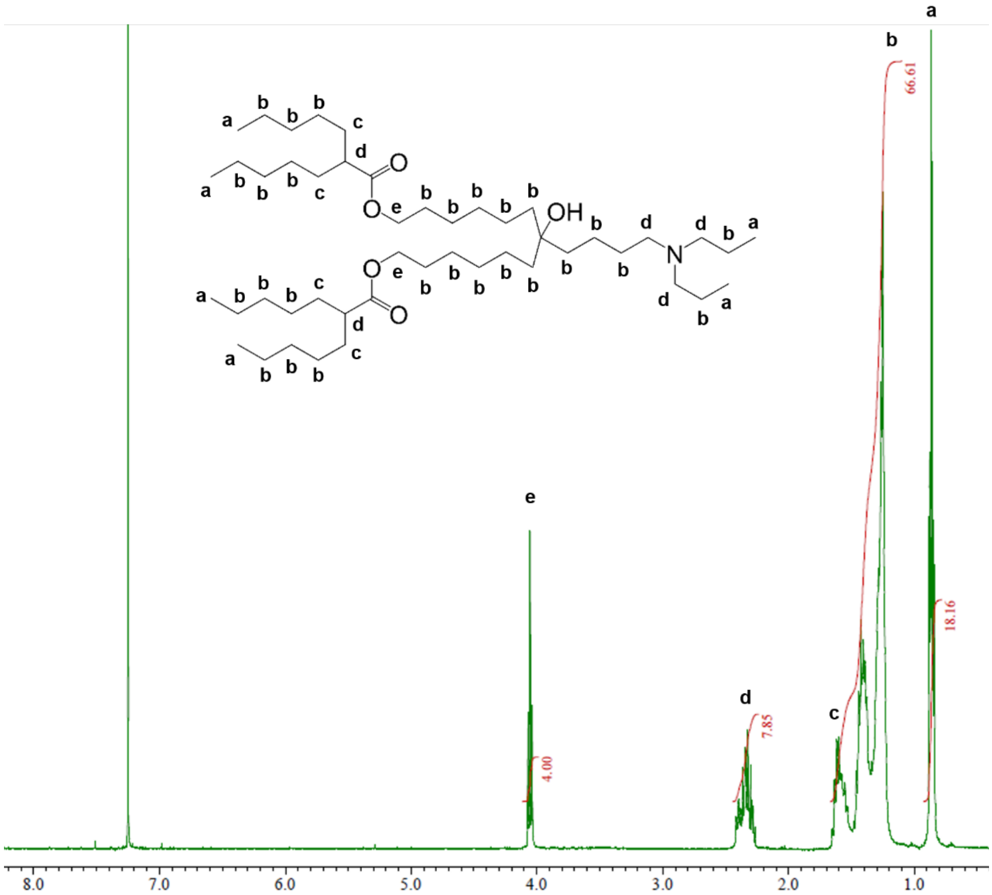

Reference

[1] R. Afshar Ghotli, A. R. Abdul Aziz, I. M. Atadashi, D. B. Hasan, P. S. Kong, M. K. Aroua, *J. Ind. Eng. Chem.* **2015**, *21*, 1039.
